# An Orally Deliverable, Food-Compatible Lyophilized Recombinant Whole-Cell Catalyst for Alcohol-Associated Liver Injury

**DOI:** 10.3390/microorganisms14040746

**Published:** 2026-03-26

**Authors:** Fan Li, Meng-Yue Zhang, Xiao-Le Shan, Cai-Yun Wang, Ying-Ying Wu, Shuang Li, Shi-Qiao Xu, Yi-Xuan Zhang

**Affiliations:** 1 School of Life Science and Biopharmaceutics, Shenyang Pharmaceutical University, 103 Wenhua Road, Shenyang 110016, China; lifanbest1@163.com (F.L.); iamzmy@126.com (M.-Y.Z.); jasonhuilok0120@163.com (X.-L.S.); jeskka@126.com (C.-Y.W.); wyy280884549@163.com (Y.-Y.W.); lishuang8729@163.com (S.L.); 2 Liaoning Beikri Biotechnology Co., Ltd., Shenyang 110031, China; n1694912685@163.com

**Keywords:** alcohol-associated liver injury, intestinal alcohol metabolism, gut microbiota, lyophilized recombinant whole-cell catalyst, gut–liver axis

## Abstract

Effective oral interventions for alcohol-induced metabolic stress and liver injury remain limited. Pre-absorptive gastrointestinal alcohol handling is gaining interest as a non-pharmacological strategy to reduce hepatic burden. In this study, we developed a formulation-integrated, food-compatible lyophilized recombinant whole-cell catalyst based on *Escherichia coli* Nissle 1917 engineered to express alcohol dehydrogenase and acetaldehyde dehydrogenase. Rather than focusing exclusively on strain-level genetic modification, the engineered cells were protected by lyophilization combined with a food-grade chitosan–alginate layer-by-layer coating, forming an artificial cell wall designed to enhance survivability during oral delivery. The formulation resisted simulated gastric acid, sodium taurocholate, and ethanol, retained enzymatic activity after storage, and demonstrated formulation stability. In alcohol-exposed mice, oral administration reduced blood ethanol and acetaldehyde levels, improved liver biochemical parameters, attenuated hepatic steatosis, and partially restored oxidative stress indicators. Integrated multi-omics analyses indicated coordinated gut-associated metabolic and inflammatory responses to alcohol and intervention, rather than a single dominant pathway. These findings provide hypothesis-generating evidence; causality remains to be established. Overall, this study demonstrates a proof-of-concept, food-compatible lyophilized recombinant whole-cell catalyst that integrates enzymatic function with formulation stability and gastrointestinal resilience, highlighting an applied, food-compatible microbial framework for exploring alcohol-related metabolic stress.

## 1. Introduction

Alcohol overconsumption is one of the most common risk factors for physical and mental health, which could harm multiple organs such as the liver, gut, and brain [1]. Most of the alcohol is absorbed in the gut and circulates through the bloodstream to the liver, where it begins to be metabolized [2]. The liver has various metabolic mechanisms for eliminating alcohol, mainly concluding the hepatocyte cytoplasmic alcohol dehydrogenase (ADH) system and the microsomal ethanol oxidizing system (MEOS) (Figure S1). Chronic alcohol exposure leads to amounts of acetaldehyde and reactive oxygen species (ROS), which contribute to oxidative stress, lipid peroxidation, and progressive liver injury [3]. These processes will eventually develop into alcoholic liver disease (ALD), which includes alcoholic steatosis, hepatitis, fibrosis, and cirrhosis [4]. At present, abstinence remains the most effective intervention, and therapeutic options directly targeting alcohol metabolism remain limited [5].

Enzyme therapeutics could provide extra catalytic activity, thereby achieving the purpose of systemic detoxification. For example, the injection of erythrocyte-encapsulated ADH and aldehyde dehydrogenase (ALDH) could enhance the rate of alcohol degradation [6]. However, the intravenous injection of exogenous enzymes has many disadvantages, including high cost, easy inactivation and immune reaction. More recently, engineered or recombinant microorganisms expressing alcohol-metabolizing enzymes have been investigated as oral experimental tools to reduce systemic alcohol burden [7,8,9,10,11]. While current studies have successfully validated the concept, they remain limited to strain-level engineering and have not adequately incorporated key considerations required for food or dietary applications, including formulation design, product stability, and gastrointestinal compatibility.

Probiotics have attracted considerable interest as dietary interventions for a variety of metabolic and inflammatory conditions, including ALD [12,13,14]. Numerous studies indicate that probiotics can influence host physiology by modulating gut microbiota composition, reinforcing intestinal barrier function, and regulating immune and metabolic responses [15,16]. However, these conventional probiotics could not directly participate in ethanol or acetaldehyde metabolism, which remains inherently limited.

With the development of synthetic biology, probiotics with customized functions can be designed according to the pathogenesis of different diseases, such as used for disease diagnosis, drug delivery, drug production, and degrading toxic metabolites [17,18,19,20]. Among them, probiotic *Escherichia coli* Nissle 1917 (ECN) has an excellent anti-inflammatory function, regulation of intestinal flora, and mature gene editing technology, which is widely used as a chassis organism for genetically engineered probiotics in treating tumors and gastrointestinal diseases [21,22,23]. Importantly, ECN exhibits translational advantages including scalable cultivation, genetic stability, and food-grade formulation compatibility, making it a promising platform for developing gastrointestinal active microbial systems.

In this study, we engineered *E. coli* Nissle 1917 to co-express alcohol dehydrogenase and acetaldehyde dehydrogenase under an arabinose-inducible system, enabling stepwise conversion of ethanol to acetate within the gastrointestinal lumen. Rather than positioning this approach solely as a genetic modification of a probiotic strain, we conceptualized and developed it as a food-compatible lyophilized recombinant whole-cell catalyst, in which enzymatic function, formulation stability, and gastrointestinal survivability are integrated design elements. To improve oral delivery and practical handling, we combined lyophilization with a food-compatible chitosan–alginate layer-by-layer coating to enhance survival under simulated gastric conditions and to support room-temperature stability. Using two mouse models of alcohol exposure, we evaluated associations between oral administration and blood ethanol/acetaldehyde dynamics, liver biochemical and histological features, and oxidative stress-related parameters. Furthermore, integrated 16S rRNA profiling, untargeted metabolomics, and hepatic transcriptomics were applied to provide systems-level, hypothesis-generating insights into gut–liver axis responses associated with intestinal ethanol handling. Collectively, this work establishes a proof-of-concept, food-compatible lyophilized recombinant whole-cell catalyst and provides an applied formulation framework for exploring microbial approaches to alcohol-related metabolic stress.

## 2. Methods and Materials

### 2.1. Construction of Recombinant Strains EAP

All the strains and plasmids utilized in this investigation are documented in Table S1. *E. coli* Nissle 1917 (ECN) was employed as a chassis organism for heterologous expression. *E. coli* K12 was a gene donor, providing alcohol dehydrogenase (*adhp*) and acetaldehyde dehydrogenase (*puuc*) genes. The plasmid pBAD-MCS acted as a gene donor, providing P_araBAD_ promoter and L-arabinose regulatory protein (araC). The fragments *araC*, P_araBAD_ promoter, *adhp*, and *puuc* were linked by overlapping PCR to obtain fragment *araC*_P_araBAD__*adhp*_ P_araBAD__*puuc*. The recombinant fragment was digested using restriction enzymes (*Xba* I and *Sac* I) and ligated into the pBBR1-MCS2 expression vector.

The recombinant plasmid was then introduced into ECN via electroporation, and positive clones were selected using standard laboratory antibiotic-based selection for strain construction and verification. The resulting recombinant strain was designated as EAP, as detailed in Table S1. Antibiotic selection was used exclusively during in vitro strain construction and was not applied during animal administration or functional evaluation.

### 2.2. Enzyme Activity Assay

The recombinant strain EAP was cultured for 12 h in Luria Bertani (LB) medium with 100 μg/mL kanamycin (Solarbio, Beijing, China, Cat# K8020) at 37 °C and shaken at 200 rpm as a seed culture. Then, 1% of the seed culture was cultivated for 4 h to reach OD_600_ to 0.5. Based on the optimal conditions determined from Figure S2, L-arabinose was added to a final concentration of 0.2% (*w*/*v*) to induce protein expression, and the culture was incubated at 37 °C and shaken at 200 rpm for 14 h. The cells were harvested by centrifugation at 8000 rpm and 4 °C for 15 min. The suspending cells were adjusted to 1 × 10^10^ CFU/mL in 50 mM Tris-HCl buffer (pH 7.4) and then lysed by ultrasonication using a VCX130PB ultrasonic processor (Sonics, Tokyo, Japan) with the following parameters: ultrasonication for 6 s, pause for 3 s, and a total net ultrasonic time of 3 min. The crude enzymes were obtained as the supernatant after centrifugation at 12,000 rpm for 20 min at 4 °C to remove cell debris.

The activities of ADH and ALDH were determined by continuous spectrophotometric assays based on monitoring the increase in absorbance at 340 nm due to the reduction of NAD^+^ to NADH. All assays were performed at 37 °C in a 3 mL reaction mixture containing 50 mM Tris-HCl buffer (pH 7.4) and 2 mM NAD^+^ (Solarbio, Cat# IN00102). The reactions were initiated by adding 100 μL of the crude enzyme extract. The change in absorbance at 340 nm was continuously recorded for 3–5 min against a blank. One unit (U) of enzyme activity was defined as the amount of enzyme that catalyzes the production of 1 μM of NADH per minute under the assay conditions. Control reactions without substrate (ethanol or acetaldehyde) were performed in parallel to correct for any non-specific background reduction of NAD^+^.

These substrate concentrations were selected to enable robust detection of enzymatic activity under experimental conditions and were used solely for analytical characterization, and do not reflect physiological ethanol levels.

### 2.3. Preparation of EAPCA

Based on the recombinant strain EAP, a lyophilized whole-cell catalyst coated with chitosan/sodium alginate was subsequently prepared and designated as EAPCA. The preparation of EAPCA followed a previously reported method [24] with slight modifications. Sodium alginate (Solarbio, Cat# INA9640; 2 mg/mL) and soluble chitosan (Biotopped, Beijing, China, Cat# 9012-76-4; 2 mg/mL) were respectively dissolved in 0.5 M NaCl solution (pH 6.0). Firstly, the EAP recombinant strain was induced to express ADH and ALDH with 0.2% (*w*/*v*) L-arabinose, and the fermentation quality was verified by enzyme activity assay. The harvested cells were resuspended in a freeze-drying protective agent (10% (*w*/*v*) trehalose (Solarbio, Cat# G8570), 0.2% (*w*/*v*) saccharose (Solarbio, Cat# IS0840), and 0.1% (*w*/*v*) glucose (Solarbio, Cat# G8150)) at a concentration of 1 mg wet bacteria/mL, incubated for 2 h at 25 °C, and then washed three times with 0.5 M NaCl solution [25]. After centrifugation (10,000 rpm, 2 min), the pellet was resuspended in soluble chitosan solution at a ratio of 2 mg wet bacteria/mL, and incubated statically for 1 h to complete the first-layer electrostatic coating, followed by washing. The cells were then resuspended in sodium alginate solution at a ratio of 2 mg wet bacteria/mL, and incubated statically for another 1 h to form the second-layer coating via electrostatic self-assembly. The final coated product was designated as Lyo-EAPCA.

### 2.4. Sample Preparation for SEM

The EAP and EAPCA samples were resuspended in 0.9% (*w*/*v*) sodium chloride solution. An appropriate amount of glutaraldehyde (final concentration 2%) was added and fixed for 6 h at 4 °C. An appropriate amount of bacterial suspension was pipetted onto a clean cover slip, spread evenly, air-dried, and then fixed chemically. The cells were immersed in 40, 70, 90, and 100% alcohol for 15 min, respectively. The specimens were placed under a scanning electron microscope (Thermo Scientific™ , Waltham, MA, USA, Apreo 2C) and photographed at the end of immersion.

### 2.5. Measurement of Zeta Potential

The appropriate amounts of samples were put into the Malvern potential sample cell, and the surface charges of different coatings were measured by an electrophoretic light scattering (ELS) system using a nano-particle size potentiometer (Malvern, Malvern, UK, Nano ZS90) according to the manufacturer’s instructions.

### 2.6. The Biodegradation of Alcohol In Vitro and In Vivo

The alcohol degradation reaction mixture consisted of 50 mM Tris-HCl buffer (pH 7.4), 2% NAD^+^, and ethanol at varying concentrations (1%, 5%, 10%, 20% *v*/*v*). The 1 × 10^10^ CFUs of EAP cell pellets were resuspended in 10 mL of the reaction mixture at each concentration and incubated at 37 °C at 150 rpm for different time intervals (0, 1, 2, 3, and 4 h). After the reaction was completed, it was lysed by ultrasonication (ultrasonication for 6 s, pause for 3 s, and a total net ultrasonic time of 3 min) and then collected supernatant by centrifugation at 10,000 rpm for 5 min, which was used for ethanol and acetaldehyde content determination. Cell lysis was performed to quantify total residual ethanol and acetaldehyde associated with enzymatic activity, serving as an analytical endpoint, rather than to simulate intact-cell gastrointestinal conditions.

To verify the degradation efficiency of the recombinant strain in vivo, eighteen male C57BL/6 mice (6–8 weeks) were divided into three groups (adapted to the environment for one week before further experiments) and gavaged with PBS, ECN (1 × 10^10^ CFU/mL), and EAP (1 × 10^10^ CFU/mL), respectively, at 10 μL/g mouse. Alcohol (5 mg/g) was given by gavage after 30 min. Blood samples (30 μL from the tail vein) were collected 1 h after administration to detect blood alcohol and acetaldehyde.

To verify the effect of the artificial cell wall (alginate–chitosan coating) on the alcohol degradation ability of the recombinant strain in vivo, eighteen male C57BL/6 mice (6–8 weeks) were divided into three groups (PBS, EAP and EAPCA group). Each mouse was first orally administered with 5 mg/g alcohol, followed 15 min later by oral administration of PBS, EAP (1 × 10^10^ CFU/mL), and EAPCA (1 × 10^10^ CFU/mL), respectively, at 10 μL/g mouse. Blood samples (30 μL from the tail vein) were collected at 0 h, 1 h, 2 h, 3 h, and 4 h after administration to detect blood alcohol and acetaldehyde. The content of alcohol and acetaldehyde was detected by an assay kit (Megazyme International, Wicklow, Ireland).

### 2.7. Resistance Analysis of EAPCA In Vitro

EAP and EAPCA were incubated in 30% alcohol, 50% alcohol, simulated gastric fluid (pH 2.5, 0.32% (*w*/*v*) pepsin), or 0.3% sodium taurocholate (contain 0.15% (*w*/*v*) pancreatin; Solarbio, Cat# YZ-110815) at 37 °C with 50 rpm shaking for 1 h. All solutions were prepared in deionized water. The starting number of different bacteria (EAP or EAPCA) was fixed at 1 × 10^9^ CFUs. Then, samples were taken at various time points, centrifuged at 9000 rpm for 5 min, and bacteria in each sample were collected, washed three times with PBS, and diluted and spread on solid agar plates containing kanamycin. After aerobic incubation at 37 °C for 48 h on LB agar medium, visible individual colonies were counted and the bacterial quantity was expressed as colony-forming units per milliliter (CFU/mL) to evaluate the protective effect of the artificial cell wall (chitosan/sodium alginate coating) on the recombinant strain EAPCA. These high alcohol concentrations were used as in vitro stress-testing conditions to evaluate extreme tolerance rather than physiological relevance.

For viability assessment, freshly prepared Free-EAP, Lyo-EAP, and Lyo-EAPCA suspensions (1 × 10^9^ CFU/mL) were frozen at −80 °C for 2 h, and lyophilized for 24 h. Post-lyophilization survival rates were determined by rehydrating the pellets with sterile PBS, performing serial dilutions, and plating on kanamycin-containing agar (50 μg/mL). Colonies were enumerated after 24 h incubation at 37 °C. Residual ADH/ALDH activity was measured according to method Section 2.2. The lyophilized samples were stored at 25 °C, with weekly measurements of viable cell counts to assess storage stability.

### 2.8. Animal Experiments

C57BL/6 male mice (6 weeks old) were bought in Liaoning Immortality Biotechnology Co., Ltd. (Shenyang, China). Experimental Unit License No.: SYXK(Liao)2021-0009. All procedures were conducted in accordance with the ARRIVE 2.0 guidelines and approved by the Institutional Animal Care and Use Committee of Shenyang Pharmaceutical University (SYPU-IACUC-S2023-0913-102). Mice were euthanized by CO_2_ asphyxiation followed by cervical dislocation.

In the mouse model of chronic and binge ethanol feeding (the NIAAA model), eighteen mice were divided into three groups (adapted to the environment for one week before further experiments), each with six mice (C57BL/6 male mice (6 weeks old)). After acclimatization, ALC and EAPCA groups were fed ad libitum with a Lieber–DeCarli liquid diet containing 5% (*v*/*v*) ethanol (Bio‑Serv, Flemington, NJ, USA, F1258SP; 35% fat-derived calories, 27.6% ethanol-derived calories) for 28 days. The Normal group received an isocaloric control diet (F1259SP, Bio-Serv). Diets were freshly prepared daily and provided at 16:00–17:00 to align with nocturnal feeding behavior. On day 15, 21, and 27, mice received a binge gavage of 31.5% (*v*/*v*) ethanol (5 g/kg body weight; prepared fresh with 95% ethanol) at 07:00–09:00 a.m. to induce peak blood alcohol levels and acute liver injury. The Normal group was gavaged with PBS. The EAPCA group was administered EAPCA at 1 × 10^8^ CFU/g body weight daily for 28 days.

Twenty-four mice were randomly assigned to four groups using stratified randomization in the high-fat diet (HFD) and alcohol-induced intoxication model, and mice were fed a high-fat diet (HFD, 60% kcal% fat; catalog no. D12492; Research Diet, Beijing HFK Bio-Technology Co., Ltd., Beijing, China) for 84 days, and the Normal group was replaced maintenance feed. The HFD group was not intragastric with alcohol, and the daily alcohol management of the HFD + ALC group and the EAPCA group was the same as above. The biochemical index (aspartate aminotransferase (AST), alanine aminotransferase (ALT), low-density lipoprotein (LDL), high-density lipoprotein (HDL), Triglycerides (TG), Total Bile Acids (TBAs), total cholesterol (T-CHO), glutathione (GSH), total superoxide dismutase (T-SOD), malondialdehyde (MDA), Total Antioxidant Capacity (T-AOC)) analysis followed the protocols outlined by the commercial kit manufacturer (NanJing JianCheng Bioengineering Institute, Nanjing, China). Sample sizes were determined based on prior studies using similar models and were sufficient to detect biologically relevant differences across treatment groups.

### 2.9. Glucose and Insulin Tolerance Tests

After intervention, the mice were used for glucose tolerance tests. The food was fasted for 8 h when water was freely available. Blood samples were collected from the tail vein of the mice and placed in the detection area of the blood glucose test strip to measure the fasting blood glucose of the mice. Then, 20% glucose solution was administered orally at a 2 g/kg dose, and blood glucose was measured at 15 min, 30 min, 60 min, and 120 min. The insulin tolerance determination method was the same, including a fasting time of about 12 h and intraperitoneal injection of insulin (Solarbio, Cat# II19102) at doses of 0.5 U/kg.

### 2.10. Histology Analysis

The mice were dissected, and the liver and colon tissue samples were collected and fixed in a 4% paraformaldehyde solution. The tissue staining with hematoxylin and eosin (H&E) was completed and entrusted to Wuhan Servicebio Technology Co., Ltd.,Wuhan, China. The histological scoring criteria for liver tissue are shown in Table S3.

### 2.11. Analysis of Short-Chain Fatty Acids

The mouse colonic contents were collected, placed in EP tubes, and frozen at −80 °C. After freeze-drying, 100 mg of sample and internal standard 2-ethylbutyric acid (final concentration 10 µg/mL) were dissolved in 1 mL 5 mM NaOH solution and centrifuged at 12,000 rpm for 10 min. An amount of 500 µL of the supernatant was then successively added with 300 µL of n-propanol, 200 µL of pyridine, and 100 µL of propyl chloroformate. Finally, 300 μL of n-hexane was added and centrifuged at 12,000 rpm for 10 min to collect the n-hexane layer into the sample bottle.

GC-MS analysis was performed by a Thermo Scientific, Waltham, MA, USA, Trace 1300-ISQ Gas Chromatograph chromatography–mass spectrometer equipped with a TG-5 MS Capillary Column (30 m × 0.25 mm, 0.25 µm). The injection port temperature was set to 260 °C, and the quantity of each sample was 1 µL. The flow rate of helium carrier gas was kept at 1.0 mL/min. The initial column temperature was set to 30 °C and held for 2 min, followed by a ramp up to 45 °C at a rate of 5 °C/min, then held for 10 min and, finally, at a 30 °C/min rate up to 200 °C and held for 3 min. The ionization was carried out in the electron impact mode at −70 eV and 300 °C. The mass spectrometry data were obtained in full-scan mode (30–200 m/z), and quantitative analysis was performed using SIM scan mode (Table S4).

### 2.12. Gut Microbiota 16S Sequencing Assay

All multi-omics analyses in this study were conducted to provide supportive, systems-level insights into biological responses associated with the intervention, rather than to establish direct causal mechanisms.

To comprehensively analyze the compositional and functional shifts in the gut microbiota, 16S ribosomal RNA (rRNA) gene sequencing was performed on cecal content samples. The experimental workflow comprised DNA extraction, library preparation, Illumina sequencing, and bioinformatic analysis as detailed below. Cecal contents were homogenized, and total microbial DNA was isolated using the Mag-Bind^®^ Soil DNA Kit (Omega Bio-tek, Norcross, GA, USA) following the manufacturer’s protocol. DNA quality and concentration were verified by 1% agarose gel electrophoresis and spectrophotometry (NanoDrop 2000, Thermo Fisher Scientific, Waltham, MA, USA). The hypervariable V3–V4 region of the 16S rRNA gene was amplified using primers 338F (5′-ACTCCTACGGGAGGCAGCAG-3′) and 806R (5′-GGACTACHVGGGTWTCTAAT-3′). Amplicons were purified using the AxyPrep DNA Gel Extraction Kit (Axygen Biosciences, Union City, CA, USA) and quantified with QuantiFluor™-ST (Promega Corporation, Madison, WI, USA). Purified amplicons were subjected to index ligation using Nextera XT Index Kit (Illumina, Inc., San Diego, CA, USA).

Libraries were pooled in equimolar ratios and sequenced on the Illumina NovaSeq 6000 platform (PE250 mode) at Beijing Biotech Pack Science Co., Ltd., Beijing, China Sequencing depth averaged 50,000 reads per sample to ensure coverage >99% (Good’s coverage index). Paired-end reads were merged using FLASH v1.2.11 with a minimum overlap of 10 bp. Quality filtering removed reads with average Phred score < 20 or length < 200 bp. High-quality sequences were clustered into Operational Taxonomic Units (OTUs) at 97% similarity using Usearch v7.0. Taxonomic assignment was performed against the SILVA database v132 via RDP Classifier v2.2 (confidence threshold: 0.7). Alpha diversity (Shannon, Simpson, Chao1 indices) was calculated using mothur v1.41. Beta diversity (Bray–Curtis distance) was visualized via PCoA and NMDS using R vegan package 2.7-3. Group differences were statistically validated by ANOSIM and Adonis tests (*p* < 0.05). Microbial metabolic functions were inferred via PICRUSt 2.0, mapping OTUs to KEGG pathways and COG categories.

### 2.13. Non-Targeted Metabolomic Analysis of Intestinal Contents

Non-targeted metabolomic profiling of cecal contents was conducted via liquid chromatography–tandem mass spectrometry (LC-MS/MS) to comprehensively characterize metabolite alterations. Samples underwent methanol-based extraction, where 300 μL HPLC-grade methanol was added to homogenized cecal content, followed by 10 min ultrasonication (40 kHz, KQ-500DE ultrasonic cleaner) at 20 °C and 30 min incubation at −20 °C. After centrifugation (12,000 rpm, 4 °C, 15 min) using a Centrifuge 5424 R (Eppendorf AG, Hamburg, Germany), supernatants were subjected to chromatographic separation using a Waters ACQUITY UPLC I-Class system equipped with a Waters HSS T3 column (1.7 μm, 2.1 × 100 mm) maintained at 40 °C. The mobile phase comprised 0.1% aqueous formic acid (A) and 100% methanol (B) with a 0.3 mL/min flow rate under a 16 min gradient: 2% B (0–1 min), ramped to 100% B (5.5 min), held (14 min), and re-equilibrated to 2% B (14.1–16 min).

Mass spectrometric detection employed a Q Exactive Plus hybrid quadrupole-Orbitrap mass spectrometer (Thermo Fisher Scientific, Waltham, MA, USA) with electrospray ionization (ESI) in polarity-switching mode. Full MS scans (m/z 150–1500) were acquired at 70,000 resolutions, with AGC targets of 1 × 10^6^ and maximum injection times of 50 MS. Data-dependent MS/MS scans (top 10 ions) utilized stepped collision energies (10, 30, 55 eV), 17,500 resolution, and AGC targets of 1 × 10^5^. Total ion chromatograms (TICs) were generated to validate analytical consistency. Raw data were processed using Progenesis QI v3.1 for peak alignment, feature extraction, and noise reduction. Metabolites were annotated against HMDB/KEGG databases with stringent thresholds: mass error < 12 ppm (MS1) or <20 ppm (MS2), retention time deviation < 0.1 min, and fragmentation scores > 45. Features detected in ≥30% of samples per group were retained; missing values were imputed as half the minimum non-zero abundance, followed by total sum normalization. Multivariate statistical analysis included Pareto-scaled principal component analysis (PCA) to evaluate group separation and orthogonal projections to latent structures discriminant analysis (OPLS-DA) to model group discrimination. OPLS-DA validity was confirmed via permutation testing (R^2^Y > 0.5, Q^2^ > 0.4), with significant metabolites identified using VIP > 1.0, *p* < 0.05 (*t*-test), and |log_2_FC| ≥ 0.585.

### 2.14. Liver Transcriptome Analysis

Hepatic transcriptomic profiling was conducted through an integrated workflow encompassing tissue processing, library construction, high-throughput sequencing, and comprehensive bioinformatic analysis. Total RNA was extracted from liver specimens using TRIzol^®^ Reagent (Invitrogen, Carlsbad, CA, USA), with stringent quality verification via Agilent 2100 Bioanalyzer (Agilent Technologies, Santa Clara, CA, USA,) confirming RNA Integrity Numbers (RINs) ≥ 7.0 and DV200 > 80%, ensuring intact mRNA for downstream analysis. Strand-specific cDNA libraries were prepared using the NEBNext^®^ Ultra™ II RNA Library Prep Kit (Illumina), incorporating poly(A) selection for mRNA enrichment followed by fragmentation at 65 °C for 5 min to disrupt secondary structures. Adapter-ligated fragments underwent PCR amplification (12 cycles) and size selection (350–450 bp) using AMPure XP beads (Beckman Coulter, Brea, CA, USA), with final library quantification via Qubit 2.0 Fluorometer and size distribution validation on an Agilent 2100 bioanalyzer.

Sequencing was performed on the Illumina NovaSeq 6000 platform (Illumina, San Diego, CA, USA; 150 bp paired-end mode), generating ~45 million reads per sample. Bioinformatic processing commenced with raw data quality control using Trimmomatic v0.39 (parameters: SLIDINGWINDOW:4:15, MINLEN:75), followed by alignment to the GRCm39 reference genome via HISAT2 v2.2.1 (–dta–rna-strandness RF), achieving >92% alignment rates. Gene expression quantification was executed with featureCounts v1.7.5 based on GENCODE vM32 annotations, calculating FPKM values to normalize for sequencing depth and gene length. Multivariate assessment included principal component analysis (PCA) revealing distinct clustering between experimental groups, while hierarchical clustering of correlation coefficients (R^2^ > 0.8) confirmed biological replicate consistency.

## 3. Results

### 3.1. Preparation and Characterization of EAPCA

**Figure 1:**
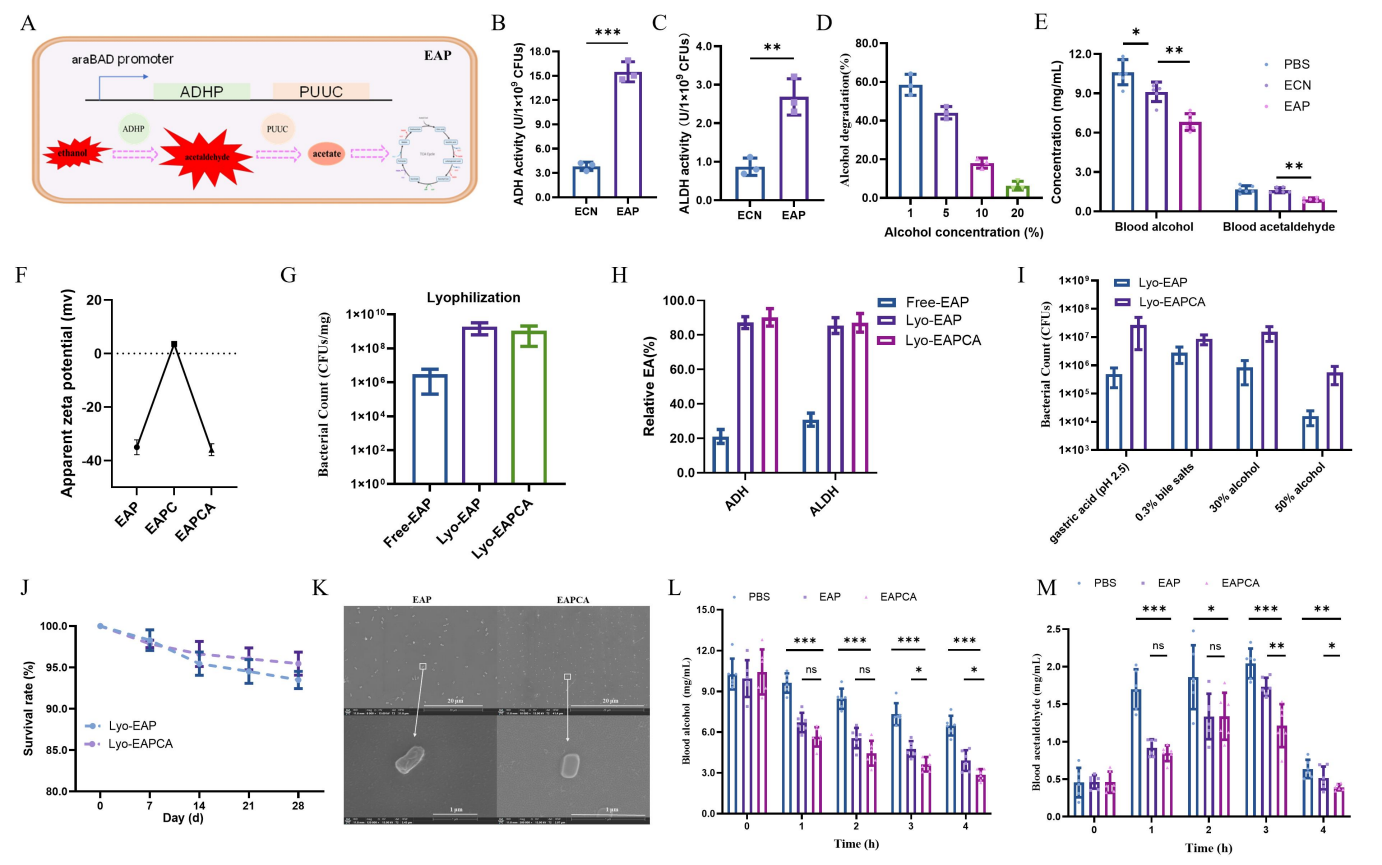
**EAPCAcharacterization of alcohol degradationability.** (**A**) Diagram of EAP. (**B**,**C**) Activity of alcohol dehydrogenase (ADH) and aldehyde dehydrogenase (ALDH) on recombinant strains EAP. (**D**) Alcohol degradation rates of the recombinant strains at different alcohol concentrations in vitro. (**E**) Alcohol concentration and acetaldehyde concentration (*n* = 6) in serum of alcohol-intoxicated mice treated with PBS, ECN, and EAP, respectively. Mice were gavaged with alcohol at 5 mg/g body weight and measured at 1 h. (**F**) Apparent zeta potential of EAP with different chitosan/sodium alginate layer coatings during the preparation of EAPCA. (**G**) Bacterial colony counts after lyophilization (freeze-drying) treatment. (**H**) Relative enzyme activity of lyophilized EAP preparations. (**I**) The colony number of EAP after treatment with 30% alcohol, 50% alcohol, 0.3% sodium taurocholate (contain 0.15% (*w*/*v*) pancreatin), and gastric acid (pH 2.5, 0.32% (*w*/*v*) pepsin). (**J**) The storage stability of EAP and EAPCA after lyophilization (freeze-drying) treatment. (**K**) SEM images of EAP and EAPCA. (**L**,**M**) Alcohol concentration and acetaldehyde concentration (*n* = 6) in serum of alcohol-intoxicated mice treated with PBS, EAP, and EAPCA, respectively. Mice were gavaged with alcohol at 5 mg/g body weight and measured at 0, 1, 2, 3, and 4 h. Data are presented as mean ± SEM. Statistical analysis was evaluated with two-tailed Student’s *t* tests (ns *p* > 0.05: no significance, * *p* < 0.05, ** *p* < 0.01, and *** *p* < 0.001).

An orally deliverable enzymatic platform was engineered for alcohol-associated liver protection, using *Escherichia coli* Nissle 1917 as an engineered probiotic vehicle expressing alcohol-metabolizing enzymes to support gastrointestinal ethanol/acetaldehyde handling in mouse models of alcohol exposure. The pBBRs-P_araBAD_ vector containing *adhp* and *puuc* genes was used to stabilize the expression of ADHP and PUUC (EAP) with the inductive agent arabinose (Figure 1A). The *adhp* and *puuc* genes were from *E. coli* K12, which converts ethanol to acetaldehyde, and the latter converts acetaldehyde to acetic acid [26]. After inducing with 0.2% arabinose, the catalytic activity of alcohol dehydrogenase (Figure 1B) and aldehyde dehydrogenase (Figure 1C) of the recombinant strain EAP was significantly increased, in which the activity of the crude enzyme was 15.49 and 2.68 U/1 × 10^9^ CFUs, respectively.

To verify the alcohol degradation ability of EAP, 1 × 10^9^ CFUs/mL bacteria were collected and incubated with different concentrations of ethanol for 6 h to determine the ethanol clearance rate. EAP degraded 44.1 ± 2.3% of ethanol at a 5% concentration (Figure 1D), with degradation kinetics plateauing beyond 6 h (Figure S3), which indicates limited additional conversion under the tested in vitro conditions. Notably, at a concentration of 10%, the ethanol degradation rate could reach 18%, which demonstrates that EAP retained measurable catalytic activity under a high ethanol concentration.

Given the dynamic physiological variables that influence oral catalytic efficiency, an in vivo model involving alcohol gavage (5 g/kg body weight) was developed. Mice exhibited typical ethanol-induced sedation after gavage, and subsequent blood analyses at the 60 min time point revealed significantly reduced alcohol and acetaldehyde concentrations in EAP cohorts versus controls (Figure 1E), indicating an association with lower systemic ethanol/acetaldehyde levels at this time point.

Lyophilization performs a pivotal technological step for achieving the stable storage and transportation of probiotics. Conventional lyoprotectants are primarily used to alleviate dehydration stress and osmotic shock during lyophilization, but they lack the capacity to protect probiotics against the low pH environment of the stomach [27]. Therefore, it is necessary to design a rational dosage form to improve the harsh environment tolerance of the recombinant strain. To achieve this, the recombinant strain was first lyophilized (freeze-dried) using appropriate cryoprotectants (10% (*w*/*v*) trehalose, 0.2% (*w*/*v*) saccharose, and 0.1% (*w*/*v*) glucose) to stabilize the protein structure and reduce the physical puncture damage to the cell membrane caused by ice crystals during the freeze-drying process [28]. Subsequently, chitosan was used as a positively charged polyelectrolyte and sodium alginate as a negatively charged polyelectrolyte to self-assemble the recombinant strain layer by layer.

Electrostatic layer-by-layer (LbL) assembly was validated by zeta potential reversal (Figure 1F), confirming sequential adsorption of cationic chitosan and anionic alginate onto EAP surfaces. The colony-forming units per milligram of freeze-dried bacteria treated with lyoprotectants (Lyo-EAP) were higher than 1.91 × 10^9^ CFUs/mg, which was significantly higher than that of the control group (Free-EAP) without lyoprotectants (3 × 10^6^ CFUs/mg), indicating the necessity of lyoprotectants during freeze-drying (Figure 1G). Without affecting the protective agent’s freeze-drying efficacy, the chitosan and sodium alginate coatings preserved bacterial viability at 1.09 × 10^9^ CFU/mg. The freeze-drying process may cause the denaturation, aggregation or chemical degradation of recombinant proteins due to ice crystal damage, dehydration stress and oxidation. In order to investigate whether the layer-by-layer self-assembled chitosan and sodium alginate would affect the protective effect of the freeze-drying protectant on the recombinant protein, the residual enzymatic activity of ADH and ALDH was measured. As shown in Figure 1H, the unprotected EAP group showed substantial activity loss, retaining only 21.03 ± 1.8% ADH and 30.83 ± 2.1% ALDH activity compared to pre-lyophilization levels. And lyoprotectant-containing groups preserved >85% residual activity for both enzymes, demonstrating that the LbL coating was compatible with the cryoprotectant formulation and did not measurably reduce residual enzyme activity after lyophilization.

### 3.2. Chitosan/Alginate Coating Enhances Lyo-Probiotic Survival in Harsh Environments

The core function of the freeze-drying protective agent is to reduce freezing damage and dehydration stress, but it cannot directly enhance the strain’s tolerance to harsh environments other than freeze-drying. Therefore, to determine whether chitosan and sodium alginate coatings can enhance the strain’s ability to resist harsh environments, the number of viable bacteria was examined after 2 h of incubation under simulated conditions of gastric acid, sodium taurocholate, and high-concentration alcohol in vitro. As shown in Figure 1I, the chitosan/alginate-coated Lyo-EAPCA significantly enhanced the strain’s resistance to stomach acid, with the number of viable bacteria being 2.67 × 10^7^ CFUs, far higher than that in the Lyo-EAP group (4.84 × 10^5^ CFUs). The chitosan/sodium alginate coating did not significantly improve tolerance to 0.3% (*w*/*v*) sodium taurocholate. Under ethanol stress conditions, Lyo-EAPCA demonstrated better survivability than Lyo-EAP, which is associated with increased tolerance in the in vitro stress-testing system. Additionally, Lyo-EAPCA exhibited stable room-temperature (25 °C) storage performance. As shown in Figure 1J, Lyo-EAPCA has better storage stability over 28 days than Lyo-EAP, with a slower decline in bacterial survival rate.

Collectively, Lyo-EAPCA demonstrates superior environmental resilience and storage stability, hereafter designated EAPCA. By observing the morphology of EAP and EAPCA through scanning electron microscopy, the results showed that chitosan and sodium alginate were adsorbed on the surface of the bacteria, forming a continuous surface coating consistent with an “artificial cell wall” (Figure 1K). To further evaluate the enhancement ability of the “artificial cell wall” to EAP, mice tail vein blood samples were collected at different time points to measure blood alcohol and blood acetaldehyde and to compare further the alcohol degradation ability of the recombinant strain coated with “artificial cell wall” (EAPCA) and the uncoated recombinant strain (EAP). During the first two hours, the blood alcohol content of EAPCA was lower than that of EAP, but there was no significant difference (Figure 1L). However, there was a substantial difference between three hours and four hours. This delayed separation is consistent with a longer duration of functional activity in vivo, although direct persistence in the gastrointestinal tract was not measured in this experiment. A significant difference was also seen in the determination of blood acetaldehyde after three hours compared with EAP (Figure 1M). In the PBS group and EAP group, the blood acetaldehyde content at three hours was higher than at two hours. This pattern suggests transient systemic accumulation of acetaldehyde during ethanol metabolism, whereas EAPCA reduced acetaldehyde levels at later time points, consistent with sustained ethanol/acetaldehyde handling capacity under the tested dosing regimen.

### 3.3. Protection Effect of EAPCA on Alcoholic Fatty Liver

**Figure 2:**
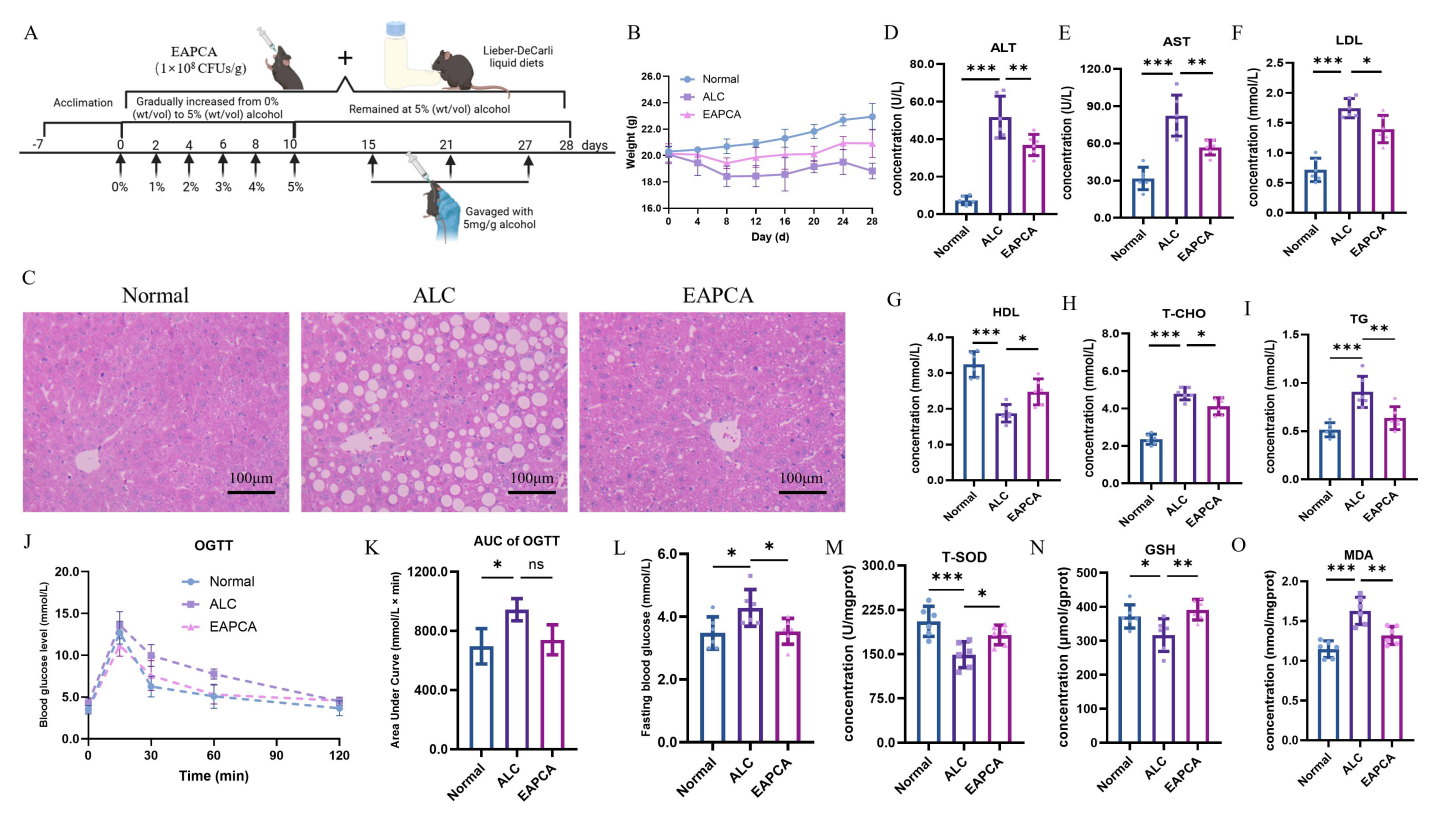
**Alleviation of alcoholic fatty liver by EAPCA intervention.** (**A**) Experimental timeline of alcohol adaptation and EAPCA intervention. (**B**) Body weight changes under chronic alcohol consumption. (**C**) The H&E staining image of different treatment group comparisons. (**D**–**I**) Serum alanine aminotransferase (ALT), aspartate aminotransferase (AST), low-density lipoprotein cholesterol (LDL), high-density lipoprotein cholesterol (HDL), total cholesterol (T-CHO), and triglyceride (TG) in liver of mice (*n* = 6). (**J**,**K**) Oral glucose tolerance tests (OGTTs) for different treatment groups (*n* = 6). (**L**) Fasting blood glucose in different treatment groups (*n* = 6). (**M**–**O**) Reduced glutathione (GSH), total superoxide dismutase (T-SOD), and malondialdehyde (MDA) in liver of mice (*n* = 6). Data are presented as mean ± SEM. Statistical analysis was evaluated with two-tailed Student’s *t* tests (ns *p* > 0.05: no significance, * *p* < 0.05, ** *p* < 0.01, and *** *p* < 0.001).

As shown in Figure 2A, alcoholic fatty liver was induced in male C57BL/6J mice through chronic alcohol exposure via Lieber–DeCarli liquid diet gradually increased from 0% to 5% (*w*/*v*) over 10 days, maintained at 5% for 18 subsequent days, and concluded with intragastric gavage of 5 mg/g alcohol on day 15, 21, and 27. The results of body weight change showed that the ALC group exhibited suppression of weight gain throughout the 28-day experimental period, while the weight of the group with EAPCA intervention plateaued at approximately 20.0 g (Figure 2B). The organ index results revealed that the liver-to-body weight ratio (Figure S4) in the ALC group was significantly higher than that in the EAPCA group, with interpretation made in conjunction with body weight differences across groups. Next, the mice’s liver tissue samples were analyzed to explore the effect of alcohol degradation in the intestine by EAPCA on relieving liver metabolic stress. H&E staining was used to evaluate the pathological changes in the liver in mice (Figure 2C and Figure S5). Compared to the Normal group, the liver tissue histopathological results of the ALC group showed obvious inflammatory cell infiltration and fat droplet accumulation. However, liver histopathological features in the EAPCA group closely resembled those of healthy controls, suggesting attenuation of alcohol-induced steatosis and inflammatory infiltration. The serum aspartate aminotransferase (AST), alanine aminotransferase (ALT), low-density lipoprotein cholesterol (LDL), high-density lipoprotein cholesterol (HDL), triglyceride (TG), and total cholesterol (T-CHO) were further detected to evaluate hepatotoxicity in vivo.

Subsequently, liver biochemical indices were analyzed to assess hepatic function. The results showed that the serum levels of alanine aminotransferase (ALT) (Figure 2D) and aspartate aminotransferase (AST) (Figure 2E) in the ALC group were significantly higher than the EAPCA-supplemented group. Serum low-density lipoprotein cholesterol (LDL) increased and high-density lipoprotein cholesterol (HDL) decreased in the ALC group, accompanied by elevated total cholesterol (T-CHO) (Figure 2H) and triglyceride (TG) (Figure 2I). These lipid changes were partially improved in the EAPCA group (Figure 2F–I). To explore the effect of alcohol on glucose metabolism in the liver, oral glucose tolerance was measured in the different groups. As shown in Figure 2J, the curve in the ALC group was higher than that in the Normal group, indicating that alcohol affected the body’s glucose tolerance. Calculating the area under the curve can effectively quantify the overall glucose handling capacity of the body. The results showed that the area under the curve value of the ALC group was significantly higher than that of the Normal group, but there was no significant difference between the ALC and EAPCA groups (Figure 2K). Subsequently, different groups’ fasting blood glucose values were measured, and the results showed that the ALC group was significantly higher than the other groups (Figure 2L).

Furthermore, the antioxidant capacity of hepatocytes was investigated. The contents of superoxide dismutase (SOD) (Figure 2M) and reduced glutathione (GSH) (Figure 2N) in the ALC group were significantly lower than those in the EAPCA group, indicating impaired antioxidant capacity in the ALC group and partial restoration with EAPCA. As shown in Figure 2O, the malondialdehyde (MDA) content of the ALC group was 1.78 times that of the EAPCA group, and the MDA content of the EAPCA group was slightly lower than that of the Normal group, indicating that EAPCA could effectively alleviate lipid peroxidation in liver tissue under these experimental conditions.

### 3.4. Regulatory Effect of EAPCA on the Gut Microbiome

**Figure 3:**
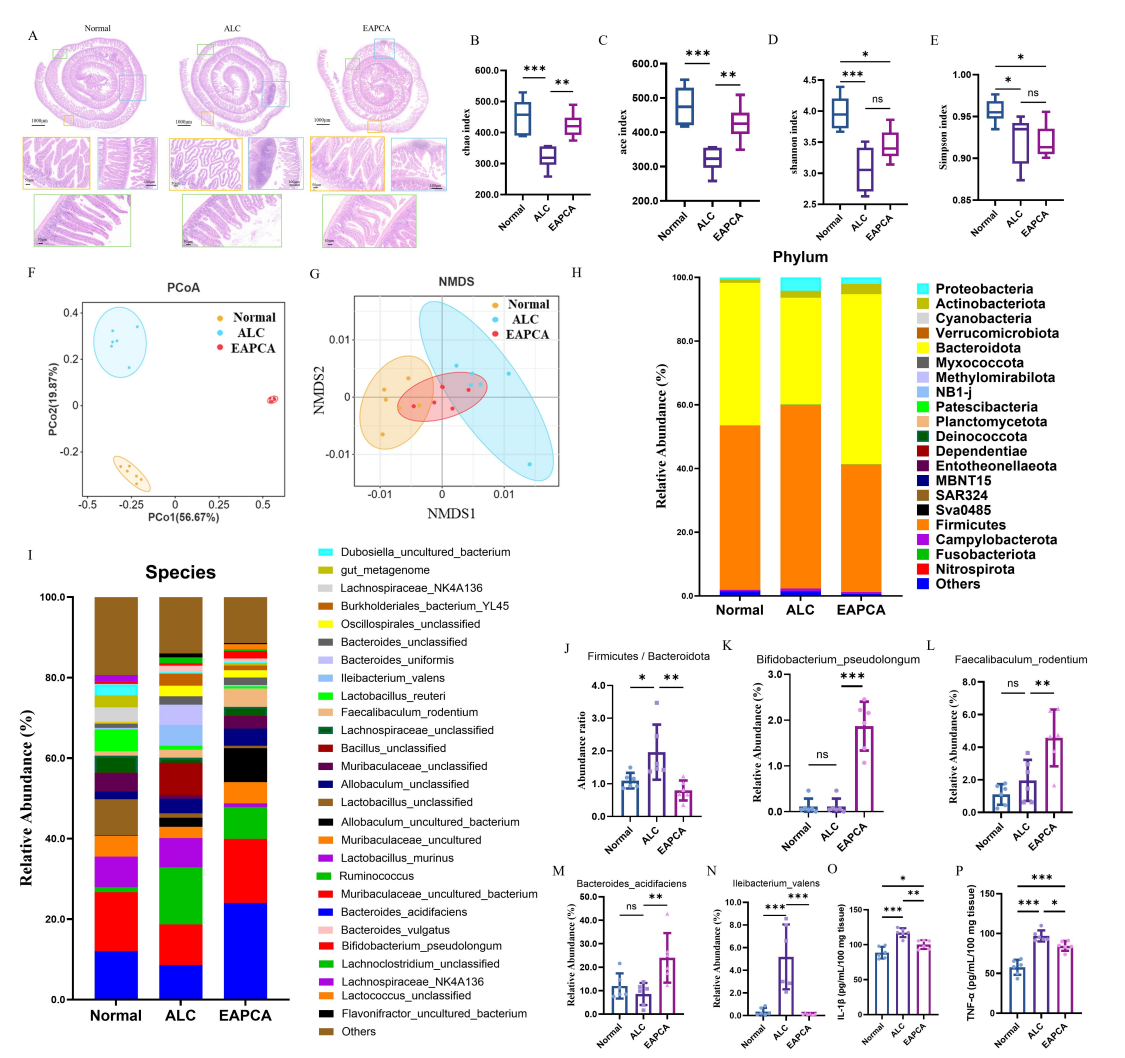
**EAPCA ameliorates alcohol-induced gut dysbiosis and inflammation through microbiota modulation.** (**A**) The H&E-stained intestinal sections of different supplemented group comparisons. (**B**–**E**) α-diversity indices Chao1 (richness), ACE (coverage), Shannon (diversity), and Simpson (dominance) of gut microbiota. (**F**,**G**) β-diversity PCoA (principal coordinate analysis) and NMDS (non-metric multidimensional scaling) analysis. (**H**) Phylum-level taxonomic composition analysis. (**I**) Species-level taxonomic composition analysis. (**J**–**N**) Statistical analysis of characteristic bacterial abundance. (**O**,**P**) Levels of pro-inflammatory cytokines (IL-1β and TNF-α) in intestinal tissue. Data are presented as mean ± SEM. Statistical analysis was evaluated with two-tailed Student’s *t* tests (ns *p* > 0.05: no significance, * *p* < 0.05, ** *p* < 0.01, and *** *p* < 0.001).

The intestinal barrier includes physical, chemical, functional, and immunological barriers [29]. Impairment of the intestinal barrier causes substances normally retained in the intestinal lumen to reach the liver through the portal circulation and stimulate inflammation. Histopathological changes in the small intestine of mice were analyzed to determine whether EAPCA contributes to improving intestinal barrier function. The Swiss-rolling technique can observe the entire intestinal mucosa condition and conduct a more extensive assessment of the intestine (Figure 3A). The experimental results show that in the yellow and green box area, the small intestinal villi in the ALC group are significantly atrophied, with widened spaces and looseness. In the EAPCA group, some of the small intestinal villi are also damaged, with widened villus spaces. In the blue box area, both the ALC group and the EAPCA-supplemented group have lymphocyte infiltration in the lamina propria and submucosa of the small intestine. Overall, intestinal histology suggested alcohol-associated mucosal injury with partial improvement in the EAPCA group, while inflammatory features remained detectable in both alcohol-exposed groups.

The gut microbiome plays an important role in host physiology, metabolism and immune homeostasis, which could interact with multiple organs through the “gut–organ axis” to form a dynamic network and affect general health [30,31,32]. To investigate the protective effects of EAPCA against alcohol-induced gut microbiota dysbiosis, we collected cecal contents and performed 16S rRNA gene sequencing analysis. This approach enabled comprehensive characterization of microbial community structure alterations following alcohol exposure and EAPCA intervention. The three groups exhibited distinct distribution patterns across all four alpha diversity indices (Figure 3B–E). In particular, the Chao and ACE indices (reflecting species richness) demonstrated more significant intergroup variations compared to the Shannon and Simpson indices (indicating species diversity and evenness), which showed relatively minor differences. The results demonstrate that alcohol significantly inhibited microbial growth, leading to decreased species richness (Chao/ACE) and diversity (Shannon). The EAPCA group displayed intermediate values between the Normal and ALC groups across all indices, suggesting partial restoration of alcohol-associated dysbiosis.

Furthermore, the ordination analyses using both principal coordinate analysis (PCoA) and non-metric multidimensional scaling (NMDS) revealed distinct patterns of separation among three different groups. The PCoA plot (Figure 3F) demonstrated clear spatial segregation, which the Normal group clustered predominantly in the lower-left quadrant, while the ALC group occupied the upper-right quadrant. Notably, the EAPCA group formed a distinct cluster isolated in the lower-right quadrant. The first two principal coordinates captured a substantial proportion of the total variation, indicating robust explanatory power for the observed group separation. However, the NMDS plot (Figure 3G) exhibited overlapping distributions, suggesting underlying gradients or shared characteristics across some group members. These ordination patterns support a measurable shift in community structure following EAPCA intervention.

At the phylum level (Figure 3H), Firmicutes and Bacteroidota were dominant. As shown in Figure 3J, the F/B ratio is significantly elevated in the ALC group. EAPCA reduced the alcohol-associated increase in the F/B ratio. Subsequent species-level analysis (Figure 3I) showed that EAPCA increased the relative abundance of Bifidobacterium pseudolongum (Figure 3K) and Bacteroides acidifaciens (Figure 3L). EAPCA also increased the relative abundance of Faecalibaculum rodentium (Figure 3M). Furthermore, the relative abundance of Ileibacterium valens was significantly elevated in the ALC group (Figure 3N). These taxonomic shifts are reported descriptively; functional implications are explored in the Discussion.

According to the 16S rRNA results, we tested whether alcohol-induced intestinal inflammation affects the short-chain fatty acid (SCFA) content. The levels of acetic acid, propionic acid, butyric acid, isobutyric acid, valeric acid, isovaleric acid, and caproic acid in different groups were determined by GC/MS. The results showed that only acetic acid significantly differed between the ALC group, Normal group, and EAPCA group (Figure S6). Furthermore, the contents of IL-1β and TNF-α in colonic cells of different groups were determined. The results showed that the two pro-inflammatory cytokines in the ALC group were significantly higher than those in the Normal group, indicating that alcohol can induce intestinal inflammation (Figure 3O,P). As expected, EAPCA intervention effectively reduced inflammatory cytokine expression, demonstrating the attenuation of alcohol-associated colonic inflammation in this model.

### 3.5. The Influence of EAPCA on the Metabolic Profile of Intestinal Contents in the Alcoholic Liver Disease

**Figure 4:**
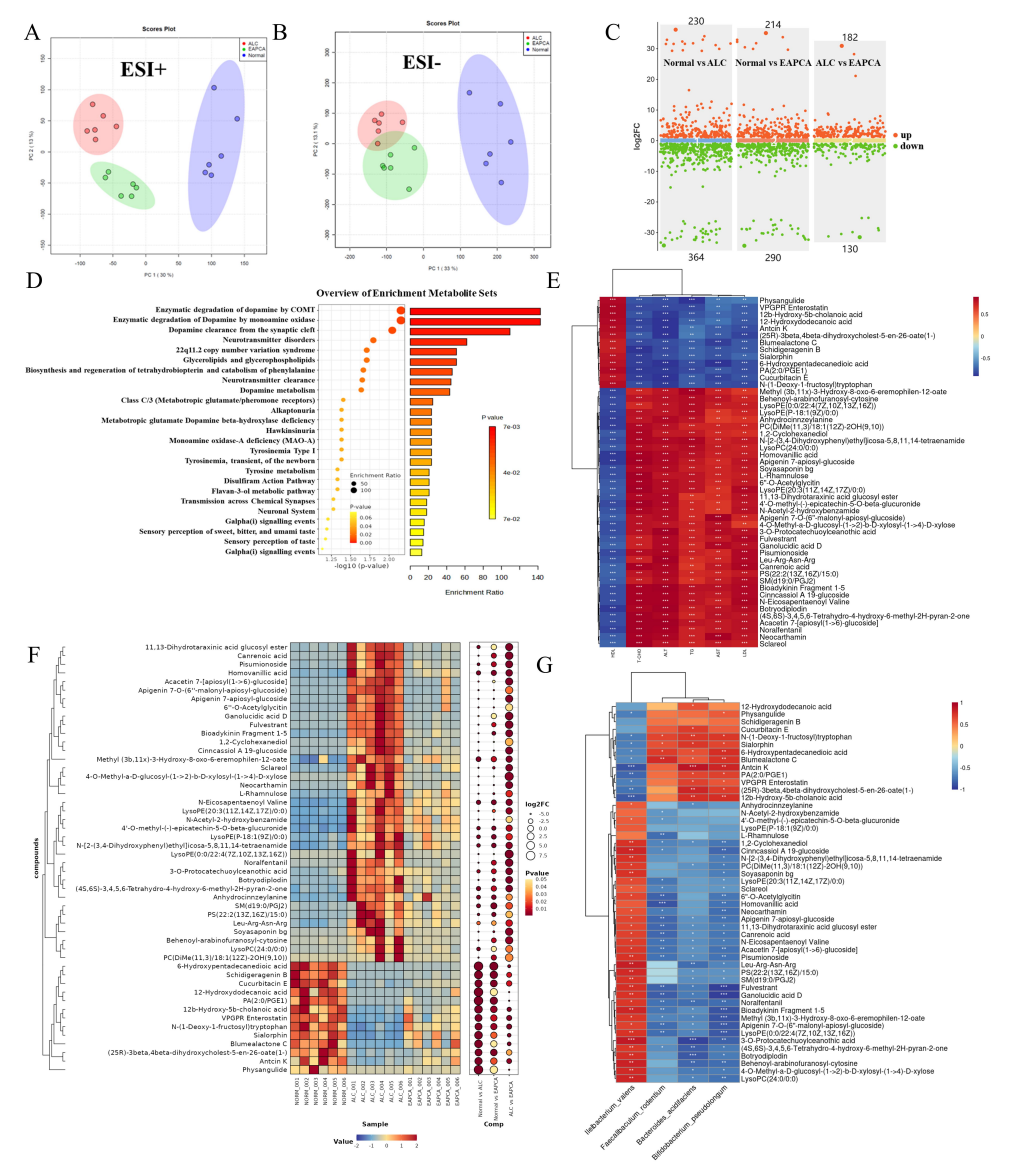
**Non-targetedmetabolomic profiling reveals significant alterations in metabolic pathways following EAPCA treatment.** (**A**,**B**) PCA score plots in ESI+ (**A**) and ESI− (**B**) ionization modes. (**C**) Volcano plot comparing −log_10_(*p*-value) and log_2_(fold change) values across different experimental conditions. (**D**) Enrichment analysis of differentially expressed metabolites between the EAPCA group and the ALC group. (**E**) Analysis of the correlation between the differential metabolites and the biochemical indicators of liver function. (**F**) Analysis of the abundance differences in metabolites in different samples. (**G**) Analysis of the correlation between the differential metabolites and the differential gut bacteria. Statistical analysis was evaluated with two-tailed Student’s *t* tests (* *p* < 0.05, ** *p* < 0.01, and *** *p* < 0.001).

In order to further explore how EAPCA affects the progression of AFLD by regulating the intestinal microecology and its metabolism, we measured the non-targeted metabolome of the cecal contents. The principal component analysis scores can illustrate the distribution of all detectable metabolites in the cecal contents in the reduced-dimensional space, thereby preliminarily exploring the overall impact of EAPCA on the cecal metabolic environment. As shown in Figure 4A,B, under both ionization modes (ESI+ and ESI−), the ALC group was significantly separated from the Normal and EAPCA groups, indicating that alcohol intervention led to a significant deviation in the cecal metabolic profile. Subsequently, a statistical analysis was conducted on the number of differential metabolites between the two groups. As shown in Figure 4C, compared with the ALC group, the Normal group showed a significant increase in 230 metabolites and a significant decrease in 364 metabolites, indicating that alcohol exposure caused a large-scale metabolic disorder. In the analysis of the differentially expressed metabolites between the ALC group and the EAPCA group, a total of 182 metabolites were upregulated and 130 compounds were downregulated.

Subsequently, the differential metabolites were subjected to pathway enrichment analysis. As shown in Figure 4D, a total of 25 pathways were enriched, which can be classified into pathways related to neurotransmitter metabolism and synaptic regulation, amino acid metabolism pathways, lipid metabolism pathways, receptor and signal transduction pathways, and others. The pathways related to neurotransmitter metabolism and synaptic transmission include the dopamine metabolism, enzymatic degradation by COMT, enzymatic degradation by MAO, dopamine clearance from synaptic cleft, neurotransmitter clearance, transmission across chemical synapses and neuronal system. The pathways related to amino acid metabolism pathways include the tyrosine metabolism, biosynthesis and regeneration of tetrahydrobiopterin and catabolism of phenylalanine. The pathways related to receptor and signal transduction pathways include class C/3 (metabotropic glutamate /pheromone receptors), galpha (i) signaling events, and sensory perception of taste (pathway names are reported as returned by the enrichment workflow).

Furthermore, correlation analysis was performed between the significantly changed metabolites and hepatic phenotypic readouts (AST, ALT, LDL, TG, and T-CHO) to explore associations between the intestinal metabolic profile and liver injury indicators. Correlation coefficients are visualized in Figure 4E using a bidirectional color scale. Metabolites showing negative correlations with liver injury indices were interpreted as metabolic features associated with a milder phenotype, whereas metabolites positively correlated with these indices were considered features associated with a more pronounced injury-related profile. In total, 13 metabolites displayed significant negative correlations with the liver injury indices, and 37 metabolites showed significant positive correlations (Figure 4E).

To avoid over-interpretation, we note that these correlation results do not establish causality and may reflect either host- or microbiota-derived metabolic shifts accompanying phenotype improvement. Among the negatively correlated metabolites, a subset of database annotations matched compounds that have been reported to influence inflammatory or metabolic signaling in other experimental systems. For example, Antcin K and Cucurbitacin E have been described to modulate inflammatory and metabolic signaling pathways in prior studies; however, their presence in our dataset should be regarded as an annotation-based observation rather than evidence of efficacy in ALD, and no therapeutic claims are made based on correlation alone. Accordingly, these metabolites are best viewed as hypothesis-generating candidates for follow-up validation (e.g., targeted quantification, source tracing, and functional testing).

The abundance patterns of the selected key metabolites across groups are summarized in Figure 4F, where the EAPCA group shows a metabolite profile closer to the Normal group than the ALC group for a subset of features, consistent with partial normalization of the intestinal metabolic environment under intervention. Because microbial functional outputs are largely mediated through metabolites, we next examined associations between differential bacterial taxa and the key metabolites (Figure 4G) to describe a microbiota–metabolite linkage under EAPCA intervention. Together, these integrated association analyses (microbiota–phenotype, metabolite–phenotype, and microbiota–metabolite) support a coherent association network, providing a rational basis for future mechanistic experiments to test whether specific microbes and metabolites contribute to phenotype modulation.

### 3.6. Transcriptome Analysis of Pathways Associated with EAPCA Intervention in Alcoholic Liver Disease

**Figure 5:**
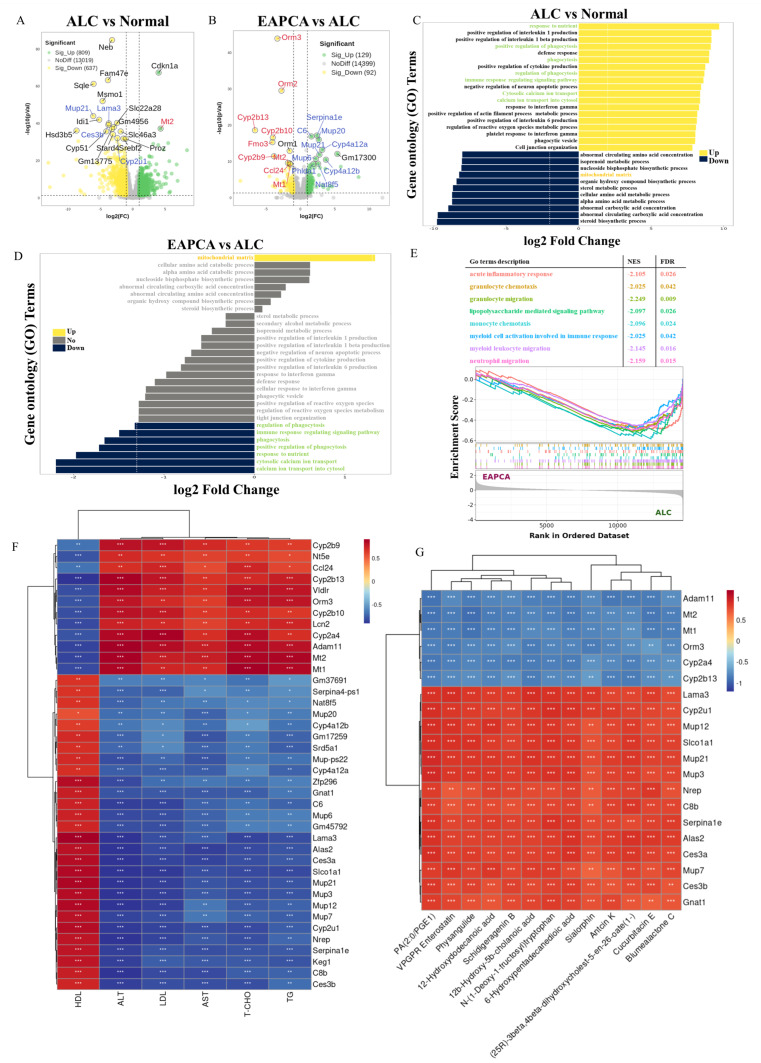
Liver transcriptomics analysis of the protective effect of EAPCA. (**A**) Volcano plot comparing −log_10_(*p*-value) and log_2_(fold change) values across ALC and Normal group. (**B**) Volcano plot comparing −log_10_(*p*-value) and log_2_(fold change) values across EAPCA and ALC group. (**C**) Enrichment analysis of differentially expressed genes between the ALC and Normal group. (**D**) Enrichment analysis of differentially expressed genes between the EAPCA group and the ALC group. (**E**) GSEA enrichment analysis of differentially expressed genes between the EAPCA group and the ALC group. (**F**) Analysis of the correlation between the differential genes and the biochemical indicators of liver function. (**G**) Analysis of the correlation between the differential metabolites and the differential genes. Statistical analysis was evaluated with two-tailed Student’s *t* tests (* *p* < 0.05, ** *p* < 0.01, and *** *p* < 0.001).

To further characterize hepatic responses, we performed liver transcriptomic profiling. Differential expression analysis identified 1446 dysregulated genes between the ALC and Normal groups (Figure 5A), including 809 upregulated and 637 downregulated genes. Upregulated genes included stress-associated transcripts such as *Cdkn1a* and *Mt2*, whereas several genes involved in lipid and sterol metabolism (e.g., *Msmo1*) were reduced in the ALC group. In addition, decreased expression of genes related to lipid metabolic processes (e.g., *Sqle*, *Idi1*, *Cyp51*) and xenobiotic transport/handling (e.g., *Slc22a28*, *Slc46a3*) was consistent with a broad transcriptional disturbance in alcohol-challenged livers, although the transcriptomic data alone do not define enzymatic activity or functional detoxification capacity.

Comparing the EAPCA-treated versus ALC samples, we identified 221 differentially expressed genes (DEGs) (129 upregulated, 92 downregulated; Figure 5B). The upregulated set included genes associated with lipid handling and ω-oxidation such as *Cyp4a12a/b*, whereas the downregulated set included inflammation-related transcripts such as *Orm1/2/3* and *Fmo3*. Collectively, these changes are associated with a shift toward altered inflammatory transcriptional signatures and partial normalization of metabolic gene programs, rather than complete restoration. Intersection analysis of DEGs shared across comparisons (Normal vs. ALC and EAPCA vs. ALC) showed that the EAPCA group displayed a more Normal-like expression pattern for a subset of genes (Figure S7), supporting a transcript-level shift toward recovery.

Gene Ontology (GO) enrichment analysis further indicated that ALC versus Normal was characterized by the enrichment of immune/inflammatory processes and suppression of metabolic pathways (Figure 5C). In the EAPCA versus ALC comparison, enriched terms included pathways related to mitochondrial organization/energetics and nutrient response, alongside reduced signatures related to phagocytosis and immune activation (Figure 5D). To complement DEG-based analysis, we performed gene set enrichment analysis (GSEA), which showed the negative enrichment of several innate immune and leukocyte trafficking terms in EAPCA versus ALC (Figure 5E), including granulocyte and neutrophil migration-related gene sets. These pathway-level results reflect coordinated transcriptional shifts rather than direct evidence of altered immune cell trafficking and warrant further validation using orthogonal approaches.

We next assessed associations between selected DEGs and liver phenotypic indicators (Figure 5F). A subset of genes (e.g., *Ces3a/b*, *Cyp4a12a/b*) showed significant negative correlations with liver injury indices, whereas others (e.g., *Ccl24*, *Lcn2*, *Cyp2b9/10/13*, *Serpina1e*, *Adam11*) were positively correlated. These associations are descriptive and do not imply causality, but they help prioritize gene candidates for follow-up validation. Finally, to explore potential links along the gut–liver axis, we examined correlations between differential intestinal metabolites and hepatic DEGs (Figure 5G). The resulting metabolite–gene association map serves as a hypothesis-generating framework to guide future mechanistic studies on how intestinal metabolic changes might relate to hepatic transcriptional responses under EAPCA intervention. No therapeutic inference is made from these associations alone.

### 3.7. EAPCA Attenuates Metabolic and Hepatic Injury Features in an HFD Plus Alcohol Challenge Model

**Figure 6:**
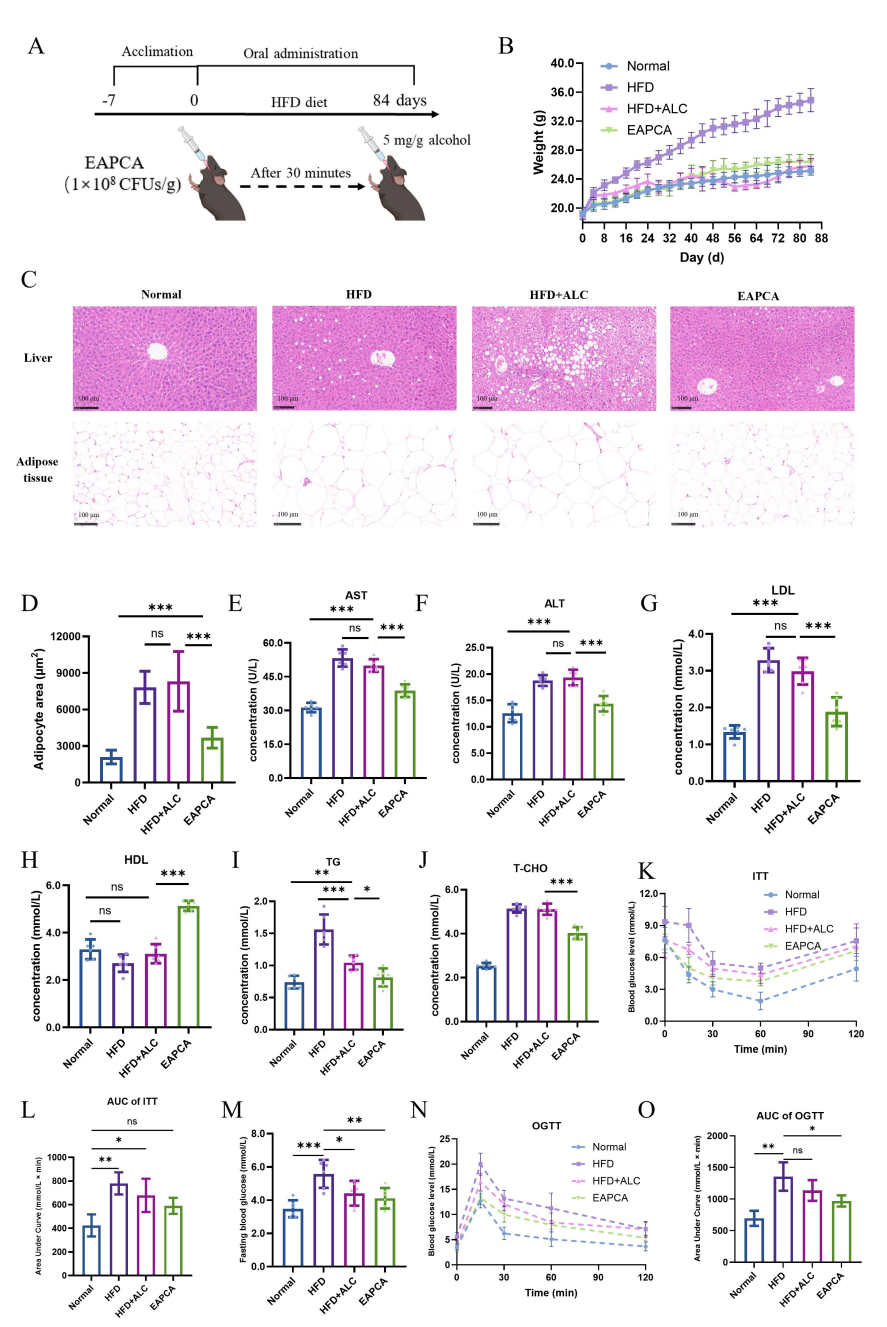
**Protective efficacy of EAPCA in HFD and alcohol intoxicated model.** (**A**) Therapeutic process of HFD and alcohol intoxicated model. (**B**) Mice body weight changes (*n* = 6) in different treatment groups. (**C**) The H&E staining image of different group comparisons. Scale bar, 100 μm. (**D**) Statistics of adipocyte area size. (**E**–**J**) Serum aspartate aminotransferase (AST), alanine aminotransferase (ALT), low-density lipoprotein cholesterol (LDL), high-density lipoprotein cholesterol (HDL), triglyceride (TG), and total cholesterol (T-CHO) in liver of mice (*n* = 6). (**K**,**L**) Insulin tolerance tests (ITTs) in different treatment groups (*n* = 6). (**M**) Fasting blood glucose in different groups (*n* = 6). (**N**,**O**) Oral glucose tolerance tests (OGTTs) for different groups (*n* = 6). Data are presented as mean ± SEM. Statistical analysis was evaluated with two-tailed Student’s *t* tests (ns *p* > 0.05: no significance, * *p* < 0.05, ** *p* < 0.01, and *** *p* < 0.001).

To evaluate EAPCA under a metabolic stress background, we used an HFD plus repeated alcohol gavage model over 84 days (Figure 6A). Body weight trajectories are shown in Figure 6B. HFD feeding induced weight gain, whereas the HFD + ALC group displayed a period of attenuated weight gain between days 28 and 56. In contrast, the EAPCA group showed a weight trajectory more comparable to HFD controls during this interval, suggesting that EAPCA administration did not further exacerbate alcohol-associated weight alterations in this model.

The liver-to-body weight ratio was increased in HFD + ALC relative to HFD (Figure S8), and this increase was partially reduced in the EAPCA group. Histological assessment (Figure 6C and Figure S9) showed that HFD + ALC livers exhibited more prominent steatosis and features compatible with hepatocellular ballooning and inflammatory infiltration, whereas the HFD and EAPCA groups displayed milder histopathological features. These observations are consistent with the biochemical readouts: serum AST and ALT were elevated in HFD + ALC and were lower in the EAPCA group (Figure 6E,F). Lipid-related indices showed a similar trend, with LDL elevated in HFD + ALC and reduced by EAPCA (Figure 6G), while HDL showed an increase under EAPCA compared with HFD + ALC (Figure 6H). TG levels were higher in HFD than HFD + ALC (Figure 6I), which may reflect complex effects of alcohol on intake, absorption, and lipid handling; therefore, TG changes are reported descriptively without directional mechanistic interpretation. T-CHO did not differ significantly between HFD and HFD + ALC (Figure 6J).

Epididymal adipose tissue analysis indicated that adipocyte hypertrophy in HFD + ALC was attenuated in the EAPCA group (Figure 6C,D), consistent with an overall shift toward improved metabolic status. To assess glucose homeostasis, insulin tolerance testing (Figure 6K,L) suggested impaired insulin responsiveness in HFD + ALC relative to HFD, while EAPCA administration was associated with a modest, partial improvement in insulin responsiveness. Fasting glucose was highest in the HFD group (Figure 6M), and EAPCA did not produce a statistically significant improvement in fasting glucose in the alcohol-challenged setting. Oral glucose tolerance testing showed group differences (Figure 6N,O), but the intervention effect was limited under the current experimental conditions and is reported accordingly. Overall, these results indicate that EAPCA is associated with the attenuation of several liver injury and dyslipidemia features in the HFD plus alcohol model, while the effects on glucose homeostasis appear modest and context-dependent.

## 4. Discussion

Oral administration of EAP represents a gastrointestinal localized approach to enable alcohol metabolism prior to systemic absorption, with the aim of reducing hepatic metabolic pressure under alcohol exposure rather than completely preventing alcohol uptake. In addition, the use of an arabinose-inducible promoter allows controlled expression of the heterologous enzymes, which provides a tunable balance between catalytic activity and host bacterial fitness. Together, these design features establish a functional evaluation framework, rather than a definitive therapeutic solution, for assessing the feasibility of an oral lyophilized recombinant whole-cell catalyst in vivo.

Lyophilized probiotics provide practical advantages for storage, transit, and long-term preservation. However, the commonly used lyoprotectants generally fail to effectively improve the tolerance of probiotics to gastric acid and sodium taurocholate. In this study, a layer-by-layer self-assembly technique was used to improve lyophilized probiotic stability throughout gastrointestinal transit. The chitosan–alginate coating provided a food-compatible and pH-responsive protective barrier, while freeze-drying protectants protected cell membrane integrity and enzymatic function during dehydration.

After oral administration of EAPCA, the blood ethanol and acetaldehyde levels were significantly reduced. Meanwhile, EAPCA intervention was linked to attenuated hepatic steatosis, improved lipid-associated indices, enhanced antioxidant capacity, and context-dependent modulation of glucose-related parameters. And these effects were observed without significant changes in body weight, suggesting that the intervention did not markedly perturb global energy balance or feeding behavior under the experimental conditions. Nevertheless, these observations reflect associative physiological improvements rather than direct evidence of therapeutic efficacy.

Alterations in gut microbiota composition represent a recognized feature of ALD. In this study, EAPCA intervention was associated with the partial restoration of gut microbial diversity, and the microbial community exhibited novel compositional characteristics. To systematically elucidate, this study performed an integrated analysis of the gut microbiota, intestinal metabolites, and hepatic gene expression, aiming to provide exploratory insights at a systems level and reveal host responses under alcohol exposure and microbial intervention. The observed transcriptional and metabolic profiles were consistent with pathways related to lipid metabolism, inflammatory signaling, and mitochondrial function, pathways that are frequently dysregulated during alcohol exposure. However, these multi-omics associations do not establish causal relationships, and the directionality of interactions along the gut–liver axis cannot be inferred from correlation analyses alone. Targeted mechanistic experiments will be required to validate specific microbe–metabolite–host links suggested by these datasets.

To address the possible constraints of plasmid expression including the risk of horizontal gene transfer to pathogenic gut flora, future research is needed to investigate alternate methodologies such as non-antibiotic selection systems, chromosomal integration, or inducible self-limiting circuits. Despite the limitations noted above, this investigation confirms the viability of a food-compatible oral whole-cell microbial enzyme platform for studying intestinal alcohol metabolism and related host reactions. Before moving forward with translational or dietary applications, additional research is needed to improve safety, validate causality, and optimize formulation.

Importantly, critical attention must be paid to the potential moral hazard associated with this technology. This lyophilized recombinant whole-cell catalyst is positioned as an auxiliary intervention for alcohol-induced intestinal and liver metabolic damage, not a “hangover cure” or a tool to excuse excessive drinking. Misuse as a “protective shield” for heavy drinking may inadvertently encourage increased alcohol consumption or mask the clinical manifestations of alcohol use disorder (AUD), which runs counter to the original intention of injury protection [33]. Therefore, this strategy must be incorporated into the comprehensive clinical management framework for alcoholic liver disease (ALD), serving only as an adjuvant intervention combined with abstinence, alcohol restriction, behavioral intervention and addiction treatment.

In summary, this study developed a food-compatible lyophilized recombinant whole-cell catalyst for pre-absorptive alcohol detoxification in the intestine. Its primary function is to chronically reduce systemic exposure to ethanol and acetaldehyde, thereby mitigating alcohol-induced liver injury. This positions the biocatalyst not as a therapeutic agent for acute alcohol intoxication, but as a preventive dietary strategy for the adjunctive management of chronic alcoholic liver disease.

## Data Availability

The data presented in this study are available on request from the corresponding author.

## Supplementary Materials

The following supporting information can be downloaded at: https://www.mdpi.com/article/10.3390/microorganisms14040746/s1, Figure S1: Schematic diagram of the key pathways of ethanol metabolism and associated pathological consequences; Figure S2: Effect of different concentrations of inducers on expression intensity; Figure S3: In vitro degradation rates of 5% alcohol by recombinant strains at different times; Figure S4: Comparison of liver organ coefficients among different groups; Figure S5: Histological scores of liver tissues from different groups; Figure S6: Short chain fatty acid content of cecal contents in different treatment groups; Figure S7: Heatmap visualization of gene expression patterns across multiple samples; Figure S8: Organ coefficient of different treatment group comparison; Figure S9: Effect of EAPCA on Histological Scores on alcohol and HFD induced liver injury; Table S1: Strains and plasmids used in this study; Table S2: Primers used in this study; Table S3: The histological scoring criteria for liver tissue; Table S4: Quantitative ions of derivatives of SCFAs.
