# Supplementary material for: An Orally Deliverable, Food-Compatible Lyophilized Recombinant Whole-Cell Catalyst for Alcohol-Associated Liver Injury
Source: Microorganisms. 2026 Mar 26;14(4):746. doi: 10.3390/microorganisms14040746 (PMC13119499; doi:10.3390/microorganisms14040746)

Table S1. Strains and plasmids used in this study

Strains and plasmids	Description
plasmids	
pX551-*gfp*	Plasmid carries *gfp* gene
pBBR1MCS-2	Broad host plasmid
pBAD-MCS	Plasmid carries P_{araBAD} promoter and L-arabinose regulatory protein (*araC*)
pBBR-*gfp*	Recombinant plasmid pBBR1MCS-2 carrying *gfp* without promoter
pBBR- *araC*_P_{araBAD}_*gfp*	Recombinant plasmid pBBR1MCS-2 carrying *gfp* initiated by P_{araBAD} promoter
pBBR-*araC*_P_{araBAD}_*adhp*-P_{araBAD}_*puuc*	Recombinant plasmid pBBR1MCS-2 carrying *adhp*, *puuc* fragment initiated by P_{araBAD} promoter
Strains	
E. coli DH5α	Providing *adhp* and *puuc* genes
E. coli Nissle 1917	Probiotics and chassis organism
ECN@ALDH	*E. coli* Nissle 1917 with plasmid pBBR-*araC*_P_{araBAD}_*adhp*-P_{araBAD}_*puuc*

Table S2. Primers used in this study

Names	Sequence (5'-3')	Enzyme site
ADHP-F	CAGGAGGAATTCACTATGAAGGCTGCAGTTGTTAC	/
ADHP-R	GGGAGTATGAAAAGTTTAGTGACGGAAATCAATCACC	/
PUUC-F	CAGGAGGAATTCACTATGAATTTTCATCATCTGGCTTACT	/
PUUC-R	C<u>GAGCTC</u>TCAGGCCTCCAGGCTTAT	Sac I
P1-F	CTAG<u>TCTAGA</u>TTATGACAACTTGACGGCTACA	Xba I
P1-R	AACTGCAGCCTTCATAGTGAATTCCTCCTGCTAGC	/
P2-F	GATTTCCGTCACTAAACTTTTCATACTCCCGCC	/
P2-R	ATGATGAAAATTCATAGTGAATTCCTCCTGCTAGC	/
Overlap-F1	CTAG<u>TCTAGA</u>TTATGACAACTTGACGG	Xba I
Overlap-R1	GGGAGTATGAAAAGTTTAGTGACG	/
Overlap-F2	GATTTCCGTCACTAAACTTTTCATAC	/
Overlap-R2	C<u>GAGCTC</u>TCAGGCCTCC	Sac I

Table S3. The histological scoring criteria for liver tissue.

Item	Definition	Score
Steatosis	Low- to medium-power evaluation of parenchymal involvement	
	<5%	0
	5 – 33%	1
	>33 – 66%	2
	>66%	3
Lobular inflammation	Overall assessment of all inflammatory foci	
	No foci	0
	<2 foci per 200× field	1
	2 – 4 foci per 200× field	2
	>4 foci per 200× field	3
Hepatocyte ballooning	None	0
	Few ballooned cells	1
	Many cells/prominent ballooning	2
Activity Score： Sum of steatosis, lobular inflammation and ballooning scores.0-8		

Table S4. Quantitative ions of derivatives of SCFAs

SCFAs	tR(min)	m/z
Acetic acid	3.67	43
Propionic acid	7.10	57
Isobutyric acid	10.04	43,71,89
Butyric acid	12.54	71
Isovaleric acid	14.87	85
Caproic acid	16.60	99
2-Ethybutyric acid	13.76	69

Figure Captions

Figure S1. Schematic diagram of the key pathways of ethanol metabolism and associated pathological consequences.

Figure S2. Effect of different concentrations of inducers on expression intensity.

Figure S3. In vitro degradation rates of 5% alcohol by recombinant strains at different times.

Figure S4. Comparison of liver organ coefficients among different groups.

Figure S5. Histological scores of liver tissues from different groups.

Figure S6. Short chain fatty acid content of cecal contents in different treatment groups.

Figure S7. Heatmap visualization of gene expression patterns across multiple samples.

Figure S8. Organ coefficient of different treatment group comparison.

Figure S9. Effect of EAPCA on Histological Scores on alcohol and HFD induced liver injury.

Figure S1.

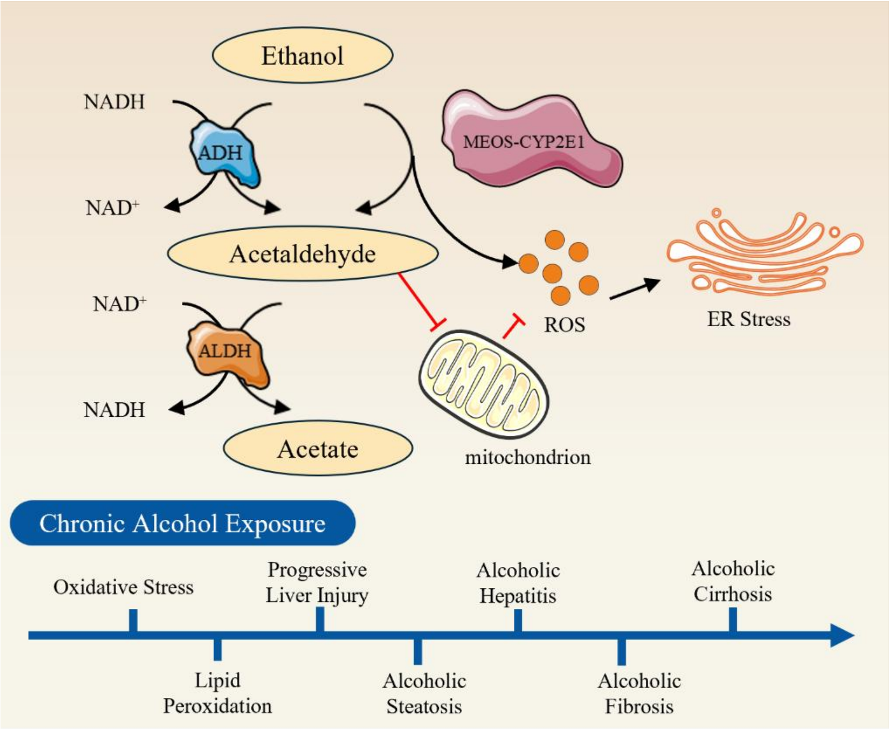
Ethanol
NADH
ADH
NAD+
MEOS-CYP2E1
Acetaldehyde
NAD+
ALDH
NADH
Acetate
ROS
ER Stress
mitochondrion
Chronic Alcohol Exposure
Oxidative Stress
Progressive Liver Injury
Alcoholic Hepatitis
Alcoholic Cirrhosis
Lipid Peroxidation
Alcoholic Steatosis
Alcoholic Fibrosis

Figure S2.

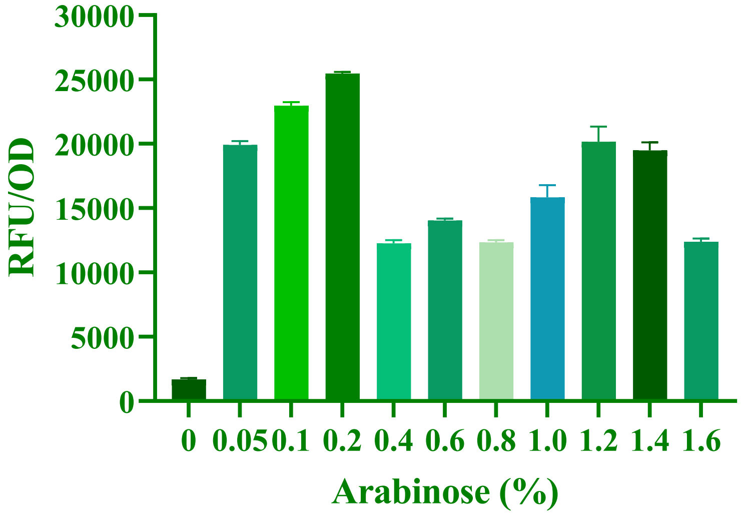
30000
25000
20000
15000
10000
5000
0
RFU/OD
0
0.05
0.1
0.2
0.4
0.6
0.8
1.0
1.2
1.4
1.6
Arabinose (%)

Figure S3.

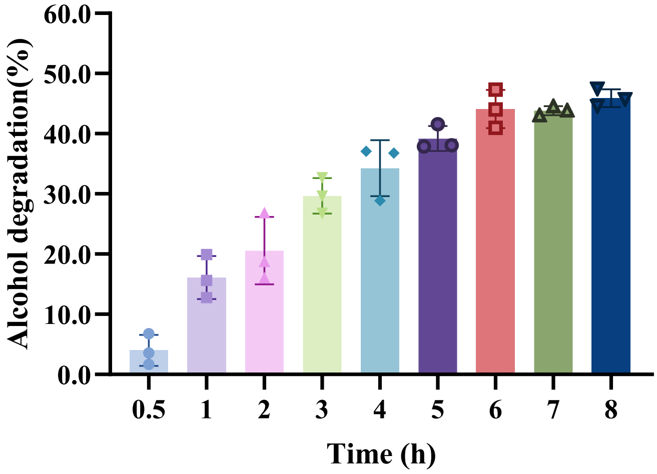
60.0
50.0
40.0
30.0
20.0
10.0
0.0
Alcohol degradation(%)
0.5
1
2
3
4
5
6
7
8
Time (h)

Figure S4.

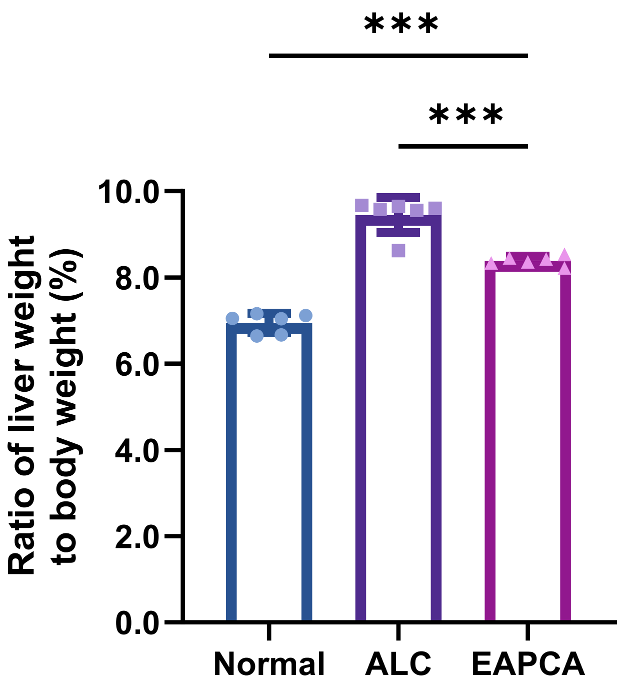

***
***
Ratio of liver weight to body weight (%)
10.0
8.0
6.0
4.0
2.0
0.0
Normal
ALC
EAPCA

Figure S5.

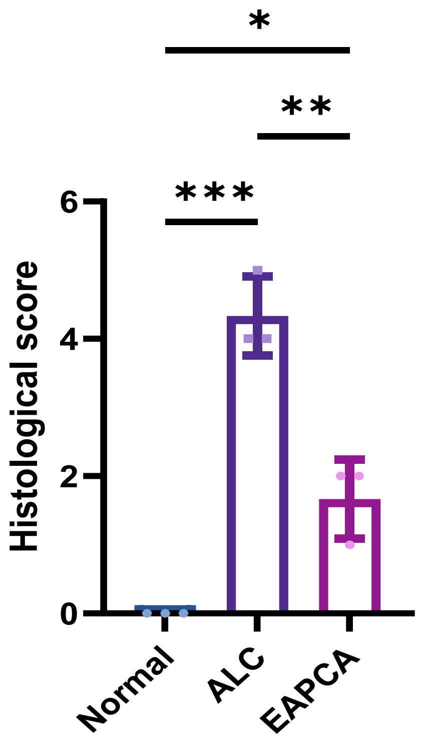

Figure S6.

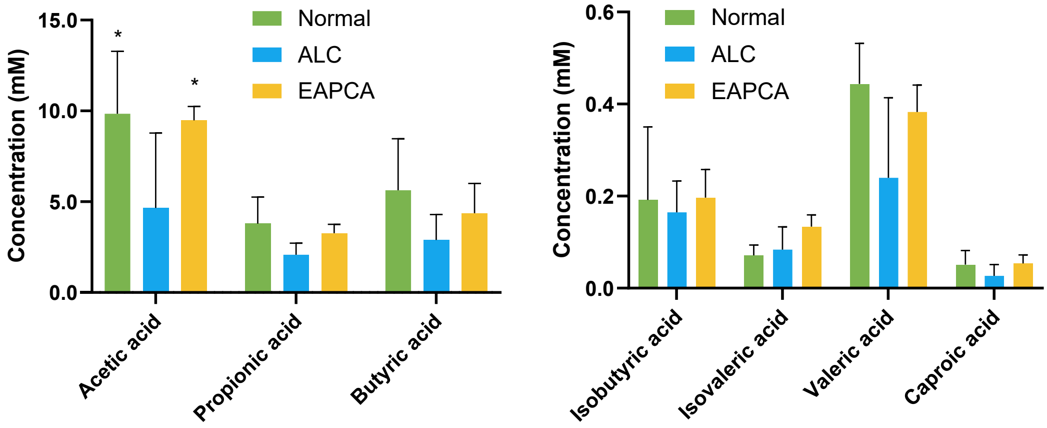
Concentration (mM)
15.0
10.0
5.0
0.0
Normal
ALC
EAPCA
*
*
Acetic acid
Propionic acid
Butyric acid
Concentration (mM)
0.6
0.4
0.2
0.0
Normal
ALC
EAPCA
Isobutyric acid
Isovaleric acid
Valeric acid
Caproic acid

Figure S7.

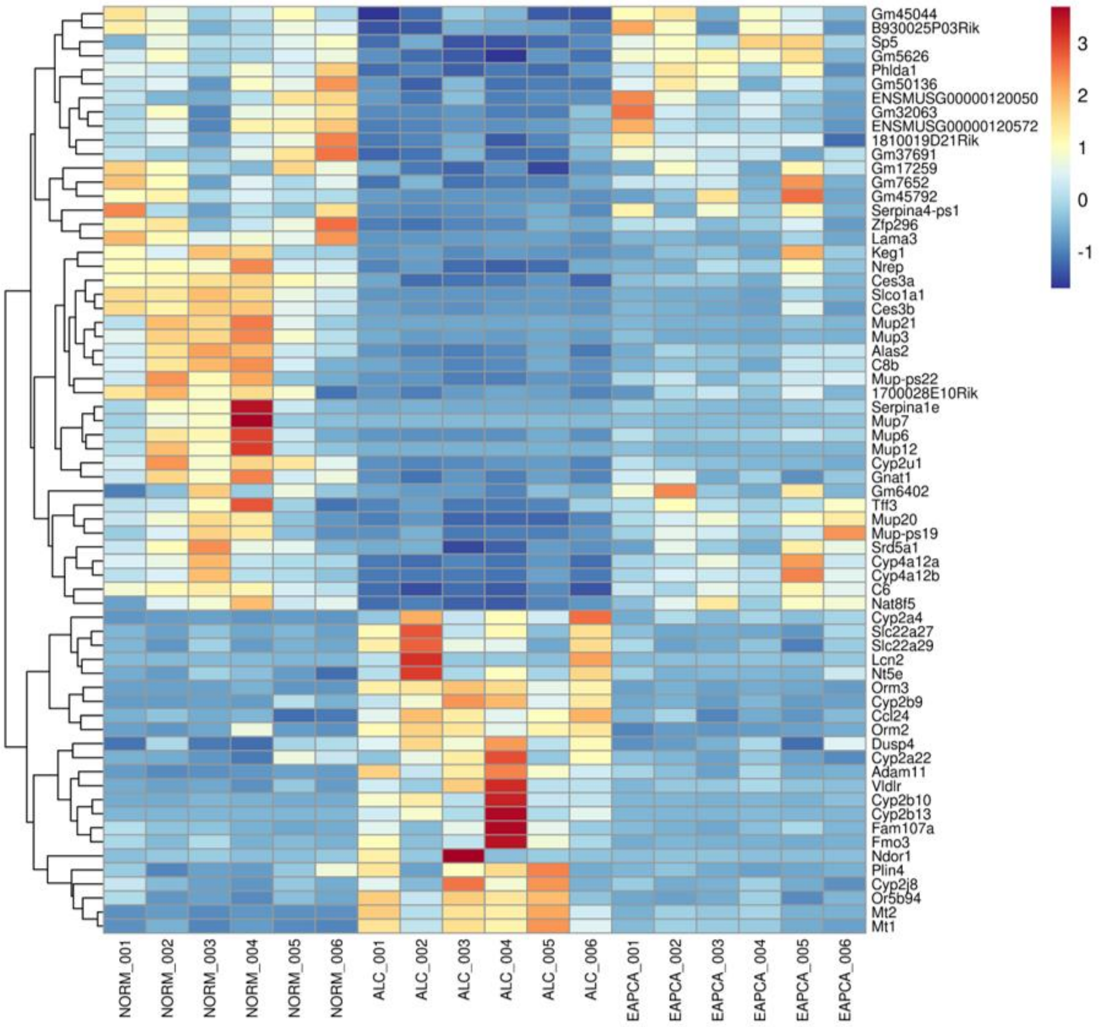
Gm45044
B930025P03Rik
Sp5
Gm5626
Phlda1
Gm50136
ENSMUSG00000120050
Gm32063
ENSMUSG00000120572
1810019D21Rik
Gm37691
Gm17259
Gm7652
Gm45792
Serpina4-ps1
Zfp296
Lama3
Keg1
Nrep
Ces3a
Slco1a1
Ces3b
Mup21
Mup3
Alas2
C8b
Mup-ps22
1700028E10Rik
Serpina1e
Mup7
Mup6
Mup12
Cyp2u1
Gnat1
Gm6402
Tff3
Mup20
Mup-ps19
Srd5a1
Cyp4a12a
Cyp4a12b
C6
Nat8f5
Cyp2a4
Slc22a27
Slc22a29
Lcn2
Nt5e
Orm3
Cyp2b9
Ccl24
Orm2
Dusp4
Cyp2a22
Adam11
Vldlr
Cyp2b10
Cyp2b13
Fam107a
Fmo3
Ndor1
Plin4
Cyp2j8
Or5b94
Mt2
Mt1
NORM_001
NORM_002
NORM_003
NORM_004
NORM_005
NORM_006
ALC_001
ALC_002
ALC_003
ALC_004
ALC_005
ALC_006
EAPCA_001
EAPCA_002
EAPCA_003
EAPCA_004
EAPCA_005
EAPCA_006
3
2
1
0
-1

Figure S8.

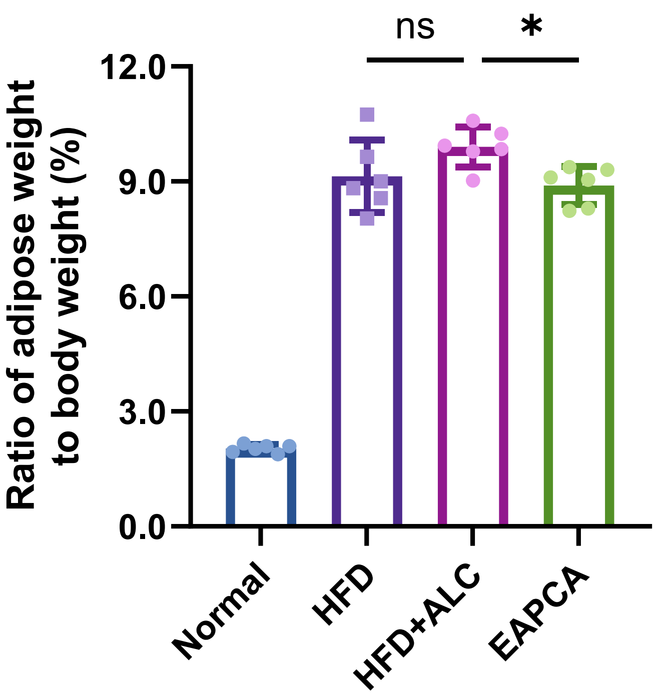
ns
*
Ratio of adipose weight to body weight (%)
12.0
9.0
6.0
3.0
0.0
Normal
HFD
HFD+ALC
EAPCA

Figure S9.

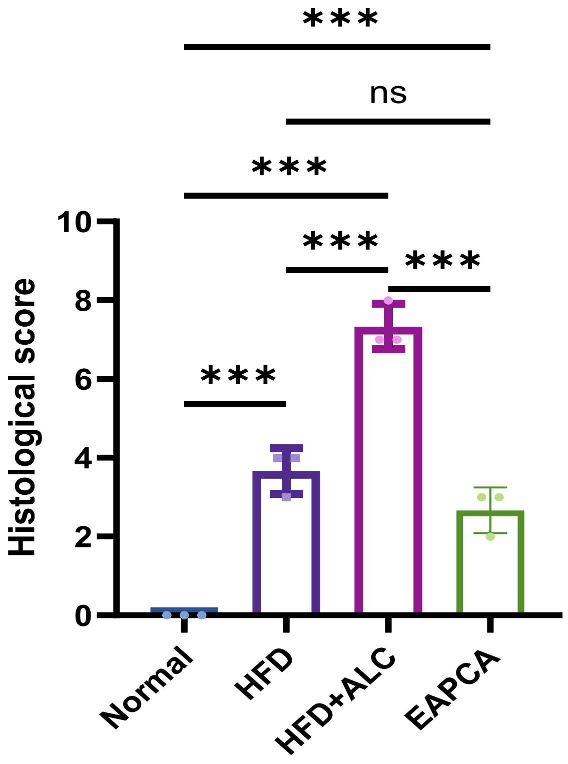

Supplement: Supplementary file 1 [file microorganisms-14-00746-s001.zip › microorganisms-4182117-supplementary.pdf]
